# Clustering and Fibril Formation during GNNQQNY Aggregation: A Molecular Dynamics Study

**DOI:** 10.3390/biom10101362

**Published:** 2020-09-24

**Authors:** Beata Szała-Mendyk, Andrzej Molski

**Affiliations:** Faculty of Chemistry, Adam Mickiewicz University, Umultowska 89b, 61-614 Poznań, Poland

**Keywords:** peptide aggregation, two-step aggregation mechanism, fibril formation, coarse-grained simulations

## Abstract

The precise kinetic pathways of peptide clustering and fibril formation are not fully understood. Here we study the initial clustering kinetics and transient cluster morphologies during aggregation of the heptapeptide fragment GNNQQNY from the yeast prion protein Sup35. We use a mid-resolution coarse-grained molecular dynamics model of Bereau and Deserno to explore the aggregation pathways from the initial random distribution of free monomers to the formation of large clusters. By increasing the system size to 72 peptides we could follow directly the molecular events leading to the formation of stable fibril-like structures. To quantify those structures we developed a new cluster helicity parameter. We found that the formation of fibril-like structures is a cooperative processes that requires a critical number of monomers, $M^{⋆}\approx 25$, in a cluster. The terminal tyrosine residue is the structural determinant in the formation of helical fibril-like structures. This work supports and quantifies the two-step aggregation model where the initially formed amorphous clusters grow and, when they are large enough, rearrange into mature twisted structures. However, in addition to the nucleated fibrillation, growing aggregates undergo further internal reorganization, which leads to more compact structures of large aggregates.

## 1. Introduction

The precise kinetic pathways of peptide clustering and fibril formation are not fully understood. One important question is whether the formation of fibrils occurs in one or two steps. The one-step model assumes that monomers aggregate directly into ordered structures. The two-step model assumes that initally amorphous clusters are formed and then, in the second step, they rearrange into ordered structures [1]. The two-step mechanism was suggested experimentally for prion and amyloid proteins [2,3]. Molecular dynamics simulations can support experiments where the microscopic information is not available [4,5,6,7].

Here, we use molecular dynamics simulations to study the initial clustering kinetics and transient morphologies during aggregation of the heptapeptide fragment GNNQQNY (abbreviated here as GNN) from the yeast prion protein Sup35. GNN is a fragment of the yeast prion protein Sup35. The Sup35 protein is able to induce misfolding and to aggregate into amyloid fibrils. The short GNN peptide is belived to drive the entire Sup35 protein to self-assemble into amyloid fibrils [8]. The GNN peptide displays the amyloid properties and aggregation kinetics similar to the full-length Sup35 protein [9]. Here, we focus on the early stage of spontaneous GNN self-assembly using long CG MD simulations of randomly distributed monomers.

Experimental studies of GNN aggregation revealed diverse morphologies of the GNN aggregates. Chiral fibrils are formed at high initial concentrations, $c_{0}>14.28$ mM [10], whereas at lower concentrations, 2.4–12 mM, crystalline aggregates are observed. Marshall and co-workers reported that the initially formed fibrils can transform into crystalline structures [11]. In our work, we explore the formation of the fibril-like structures during GNN aggregation. We study the aggregation process at the molecular level for the concentrations $c_{0}=$ 4–35 mM close to the experimental ones.

The thermodynamics and kinetics of GNN aggregation were studied with both all-atom (AA) models [12,13,14,15,16,17,18,19] and coarse-grained (CG) models [20,21,22,23,24]. The AA simulations of GNN aggregation were performed for small systems and short simulated times. Longer simulations for larger systems, up to 20 peptides, were performed using CG models. Those studies showed that even small GNN clusters may have ordered morphologies, however, the studied system were too small to aggregate into fibril-like structures.

We use a CG model developed by Bereau and Deserno [25] (referred to here as the BD model). This model has an intermediate resolution level CG force field and does not assume a particular secondary structure. The BD model was used by Osborne et al. to investigate the thermodynamics of structural transitions during the aggregation of GNN [23] and by Luiken and Bolhuis to study the primary nucleation events for GNN. Recently, the BD model was applied to study the aggregation kinetics of polyglutamine [26].

The purpose of this work is to gain further insight into the early-stage of short peptide aggregation. To this end we simulated the GNN aggregation with the BD model for systems made up of $N_{0}=20$ and 72 monomers at different initial concentrations. By increasing the system size to $N_{0}=72$, we could follow directly the molecular events leading to the formation of stable fibril-like structures. To characterize the structure emerging fibrils, we introduced a helicity parameter, *H*, and found that the newly formed clusters rearrange. The formation of fibril-like structures is a cooperative processes that requires a critical number of monomers in a cluster. We could see fibril-like structures only for the clusters above the critical aggregate size $M^{⋆}\approx 25$. For our small $N_{0}=20$ system, ordered aggregates were never formed. In the concentration range $c_{0}=$ 4–35 mM, the aggregation kinetics are consistent with the two-step aggregation model where the initially formed amorphous clusters grow and, when they are large enough, rearrange into stable twisted structures.

The structural analysis of GNN clusters and fibrils showed a special role of the tyrosine residues that form the core of cluster and fibrils. To further clarify this issue, we mutated GNNQQNY into the heptapeptide GNNQQNA (denoted here as Y7A) where the tyrosine in GNN is replaced by alanine. We found that the mutation changes significantly both the aggregation kinetics and cluster and fibril structures, which confirms the structural role of GNN tyrosine.

## 2. Methods

### 2.1. Simulations

The BD model is a generic mid-resolution, implicit solvent CG model, where each amino acid is represented by three or four beads: Three for the backbone and, for nonglycine residues, one for the side-chain. The model includes the back-bone hydrogen bonds and dipole interactions, and is parameterized such that both the α-helix and β-strand structures are accessible. This physics-based model is not biased towards a particular secondary structure nor it forces a fixed structure. Here we used an implementation of the BD model in the Espresso molecular dynamics package [27]. The software is freely available at https://github.com/tbereau/peptideB.

In the Espresso package the length, time, and mass units can be chosen by the user and then all remaining units are derived. In the BD model the length unit is 1 Å. The energy unit is $k_{B}T_{\mathrm{ref}}\approx 4.1\times 10^{-21}$ J ≈0.6 kcal mol${}^{-1}$, where the reference temperature $T_{\mathrm{ref}}=300$ K and the Boltzmann constant $k_{B}\approx 1.38\times 10^{-23}$ J K${}^{-1}$. The temperature $T_{\mathrm{ref}}=300$ K is a standard Espresso temperature and this values has been used recently in a study of polyglutamine aggregation [26]. All beads have the same mass $4.6\times 10^{-26}$ kg and the mass of a single BD bead is the mass unit. The derived time unit is τ≈0.16 ps.

The GNN simulations with the BD model were performed for two systems sizes, $N_{0}=$ 20 and 72 monomers, at four concentrations each, $c_{0}=$ 4, 8, 15, and 35 mM. We varied the concentration $c_{0}$ by keeping constant the number of monomers, $N_{0}$, and changing the simulation box size (see Table 1). The simulations were carried out using the Langevin dynamics with a time step of 0.01τ. The friction coefficient was set to $\tau^{-1}$. Each simulation was $10^{9}$ steps long and was repeated four times.

Simulations with the BD model were also performed for a mutant peptide GNNQQNA (Y7A) where the tyrosine in GNN is replaced by alanine. These simulations were performed for $N_{0}=72$ at one concentration, $c_{0}=15$ mM. Four independent repeats were conducted.

To calibrate the time unit τ of the BD model we carried out the atomistic OPLS-AA simulations for $N_{0}=20$ monomers randomly distributed in a box of size 13.05 nm, containing about 73270 TIP3 water molecules. This corresponds to the initial free monomer concentration $c_{0}=15$ mM. By comparing the monomer decay trajectories for the BD and OPLS-AA models for $N_{0}=20$ we found that, effectively, the coarse grained time unit τ≈1 ps, see Section S1 of Supplementary Materials. By this metric, each BD simulation corresponds to 10 μs. Because of the ambiguity of assigning a clock time value to τ, we express the simulation time in units of τ. Further comparison of the OPLS-AA and BD simulations for GNN is presented in Section S2 of Supplementary Materials.

### 2.2. Data Analysis

#### 2.2.1. Cluster Analysis

We refer to a free peptide molecule as a monomer. We refer to an aggregate made up of two or more peptides as a cluster. A peptide belongs to a cluster when the center of any of its atoms is less than a cut-off distance of 0.5 nm from the center of an atom of another peptide in that cluster.

Due to the periodic boundary conditions, a cluster that crosses a boundary gets split and is represented in the simulation box as two or more split clusters. To properly calculate the cluster descriptors, these split clusters are merged into a single clusters before a descriptor is calculated.

To analyze the peptide aggregation we used several descriptors: $N_{m}$—the number of free monomers; $N_{c}$—the number of clusters (dimers, trimer, and higher order aggregates); *M*—the size of a cluster, i.e., the number of peptides in the cluster; $R_{g}$—the radius of gyration of a cluster; *b*—the asphericity of a cluster defined as $b=\lambda_{z}^{2}-\left(\lambda_{x}^{2}+\lambda_{y}^{2}\right)/2$ where $\lambda_{x},\lambda_{y},\lambda_{z}$ are the principal moments of the gyration tensor and the axes are chosen such that $\lambda_{x}^{2}\leq \lambda_{y}^{2}\leq \lambda_{z}^{2}$; β—the beta-content defined as the number of residues with any beta conformation divided by the total number of peptides; and $C_{n}$—the end-to-end correlation parameter of a cluster defined as [28]

(1) $$C_{n}=\frac{2}{M(M-1)}\sum_{i<j}{\left(n_{i}\cdot n_{j}\right)}^{2}$$

where the unit vector $n_{i}$ is the normalized end-to-end vector for the peptide *i* backbone atoms. The correlation parameter $C_{n}$ is normalized in such a way that for uncorrelated $n_{i}$ vectors the parameter assumes the value 1/3 whereas for correlated $n_{i}$-s it tends to 1.

Additionally, we have developed two new descriptors of fibrillar structures: The ribbon helicity $H_{R}$ and the fibril helicity *H*.

#### 2.2.2. Ribbon Helicity, $H_{\mathrm{rib}}$, and Cluster Helicity, *H*

In our simulations large GNN clusters (fibrils) are composed of sub-units that resemble ribbons. In order to describe quantitatively the fibrillar structure of a cluster, we first determine the cluster axis, then assign the peptides to ribbons and calculate the helicities, $H_{r}$, of individual ribbons, and finally average the ribbon helicities to get the cluster helicity, *H*.

In our helicity analysis each peptide in a cluster is represented by a point. Those points are either the mass centers of entire peptides or, when the cluster axis is determined, only the mass centers of tyrosine residues. We found that the latter representation (i.e., only tyrosine residues) gives better stability when the linear regression of the points representing peptides is used for the calculation of the cluster axis.

To assign the peptides to separate ribbons, the peptide mass centers, $c_{i}$, are projected on the cluster axis to give the projections $x_{i}$. The peptides are sorted according to the positions of those projections along the cluster axis. Two distances are calculated for each pair of consecutive (sorted) peptides: $d_{\|}$ is the distance between the mass center projections along the cluster axis and $d_{⊥}$ is the distance between the projections on the plane perpendicular to the cluster axis. Two consecutive peptides belong to one ribbon if $d_{\|}\leq d_{\|}^{0}\left(M\right)$ and $d_{⊥}\leq d_{⊥}^{0}\left(M\right)$, where $d_{\|}^{0}\left(M\right)$ and $d_{⊥}^{0}\left(M\right)$ are the cluster size-dependent cut-offs. This distance criterion is based on the observation that two consecutive peptides in a ribbon are closer to each other that to any nearby peptides in other ribbons in the fibril.

A complicating factor for the helicity analysis is that the cluster structure changes with the cluster size *M*. For small cluster the distances between peptide mass centers are larger then those for larger aggregates. We found that the position $d_{m2}$ of the second maximum on the histogram of mass center distances is a good representation of the structural changes for fibrillar clusters. The second maximum changes linearly with the inverse cluster mass 1/M, $d_{m2}\left(M\right)=a/M+b$, where *a* and *b* are constants. For $N_{0}=72$ we have a=9.6192 nm and b=−0.0009 nm, see Section S3 of Supplementary Materials.

The longitudinal $d_{\|}^{0}\left(M\right)$ and vertical $d_{⊥}^{0}\left(M\right)$ cut-offs are constructed from the fit function $d_{m2}\left(M\right)=a/M+b$ and the scaling factors $s_{\|}$ and $s_{⊥}$ as $d_{\|}^{0}\left(M\right)=s_{\|}\times d_{m2}\left(M\right)$ and $d_{⊥}^{0}\left(M\right)=s_{⊥}\times d_{m2}\left(M\right)$, where the scaling factors separate the longitudinal ‖ and vertical ⊥ contributions. In the present case $s_{\|}=0.88$ and $s_{⊥}=0.47$, $s_{\|}^{2}+s_{⊥}^{2}=1$. Those values lead to the best correlation between the automatic and visual ribbon assignments.

The helicity (twist) $H_{\mathrm{rib}}$ of a ribbon is defined as the average

(2) $$H_{r}=\frac{1}{M_{\mathrm{rib}}-1}\sum_{\mathrm{ribbon}}h_{i,i+1}$$

of the contributions

(3) $$h_{i,i+1}={\hat{g}}_{i}\cdot {\hat{e}}_{i+1}$$

from peptide pairs (i,i+1) along the ribbon, where $M_{\mathrm{rib}}$ is the ribbon size. The unit vectors ${\hat{g}}_{i}$ and ${\hat{e}}_{j}$ are defined as

(4) $${\hat{g}}_{i}=\frac{{\vec{c}}_{i}-{\vec{x}}_{i}}{|{\vec{c}}_{i}-{\vec{x}}_{i}|}$$

and

(5) $${\hat{e}}_{j}=\frac{{\vec{c}}_{j}\times \hat{A}}{|{\vec{c}}_{j}\times \hat{A}|}$$

where $\hat{A}$ is a vector along the cluster axis, ${\vec{c}}_{i}$ and ${\vec{c}}_{j}$ is the vector pointing to the mass centers of peptides *i* and *j*, and ${\vec{x}}_{i}$ is the vector pointing to the projection of the mass center of peptide *i* on the cluster axis.

The helicity *H* of the whole cluster is defined as the average helicity of all ribbons in the cluster:

(6) $$H=\frac{1}{n_{\mathrm{rib}}}\sum_{\mathrm{ribbons}}H_{\mathrm{rib}}$$

where $n_{\mathrm{rib}}$ is the number of ribbons in the cluster.

## 3. Results

### 3.1. Clustering Kinetics

To study the clustering kinetics of GNN, we performed CG simulations for two system sizes $N_{0}=20$ and 72 at four concentrations $c_{0}=$ 4, 8, 15, 35 mM. For all simulated concentrations, we observed aggregation leading to the formation of clusters. We did not observe nucleation or induction periods. The aggregation was faster for larger monomer concentrations. There are two major types of elementary aggregation events: (a) monomer addition and monomer subtraction, and (b) cluster coalescence. The aggregation trajectories shows that there are three stages of aggregation. In the first stage the monomers quickly combine to form dimers that grow by monomer addition until the number of available monomers drops substantially. In the second stage the dominant process is the coalescence of clusters. Clusters may also break into smaller clusters. In the third stage large clusters undergo an internal restructuring.

**Figure 1:**
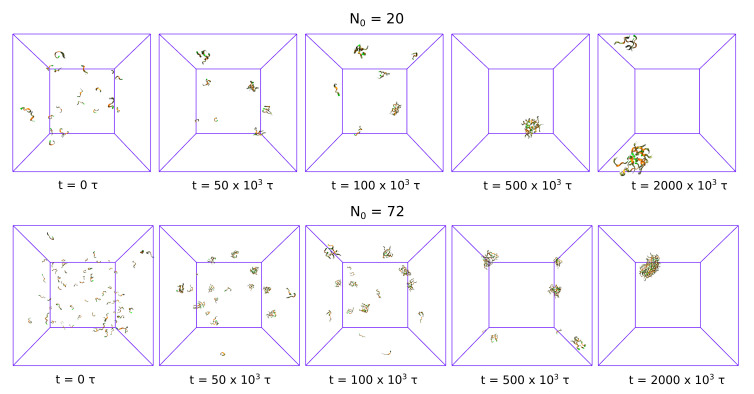
Snapshots from two simulations of GNN aggregation with the BD model. Top panel shows the small system, $N_{0}=20$, and the bottom panel shows the snapshots for the large system, $N_{0}=72$. The peptide concentration is $c_{0}=15$ mM.

Figure 1 shows simulation snapshots for two system sizes $N_{0}=$ 20 and 72 at the concentration $c_{0}=15$ mM. The simulations start with free monomers, randomly distributed in the simulation box (t=0τ). Then, the monomers aggregate and form small clusters (dimers and trimers at $t=50\times 10^{3}\tau$). The growth of clusters leads to the formation of larger aggregates and the monomer depletion ($t=100\times 10^{3}\tau$ for $N_{0}=20$ and $t=500\times 10^{3}\tau$ for $N_{0}=72$). The largest clusters, formed by coalescence of smaller clusters ($t=2000\times 10^{3}\tau$), do not split till the end of simulations.

**Figure 2:**
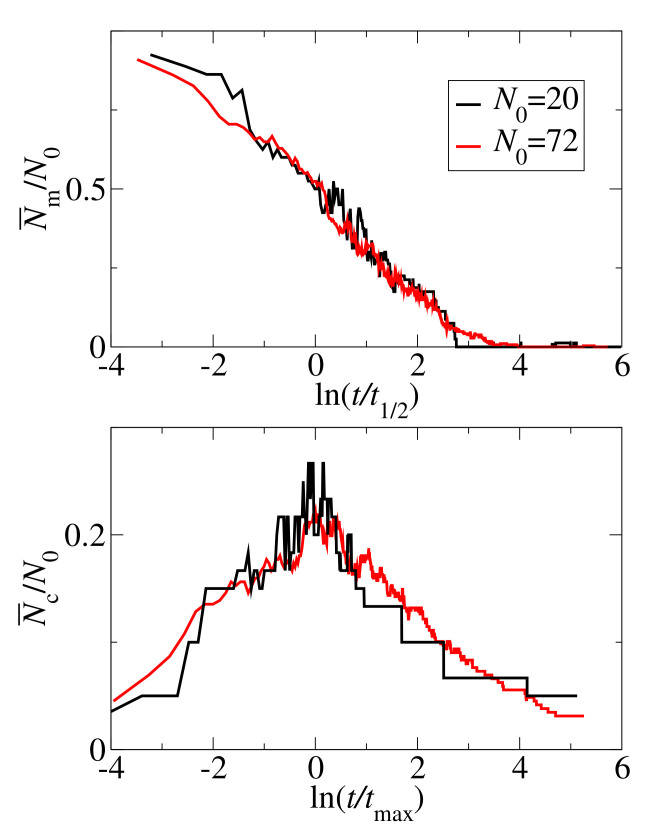
Scaled monomer (**top panel**) and cluster (**bottom panel**) kinetic curves for two system sizes, $N_{0}=20$ (black lines) and N=72 (red lines), at the concentration $c_{0}=4$ mM. The number of monomers, $N_{m}$, and number of clusters, $N_{c}$, are scaled by the initial number of peptides $N_{0}$. For the monomer decay curve, time is scaled by the half-time $t_{1/2}$. For the cluster curves, time is scaled by $t_{\max}$ defined as the time when the number of clusters reaches its maximum value. Note the logarithmic time scales.

To investigate the system size effect on the GNN aggregation, we compared the monomer and cluster kinetic curves. Figure 2 shows the scaled kinetic curves for two system sizes $N_{0}=$ 20 and 72 at the concentration $c_{0}=4$ mM. Both the number of clusters and number of free monomers are scaled by the initial number of peptides, $N_{0}$. For the monomer curves, time is scaled by the the half-time, $t_{1/2}$. For the cluster curves, time is scaled by $t_{\max}$ defined as the time when the number of clusters reaches the maximum value. For both, the monomer and cluster kinetics, the scaled curves are similar for the two system sizes. The differences are caused by the statistical noise. However, some differences are visible for the higher concentrations 8, 15 and 35 mM, see Section S4 of Supplementary Materials). Especially, the clusters plots are shifted for concentrations 15 and 35 mM. In this connection we note that, as the aggregation occurs rapidly at high concentrations, it is difficult to determine $t_{\max}$ precisely.

**Figure 3:**
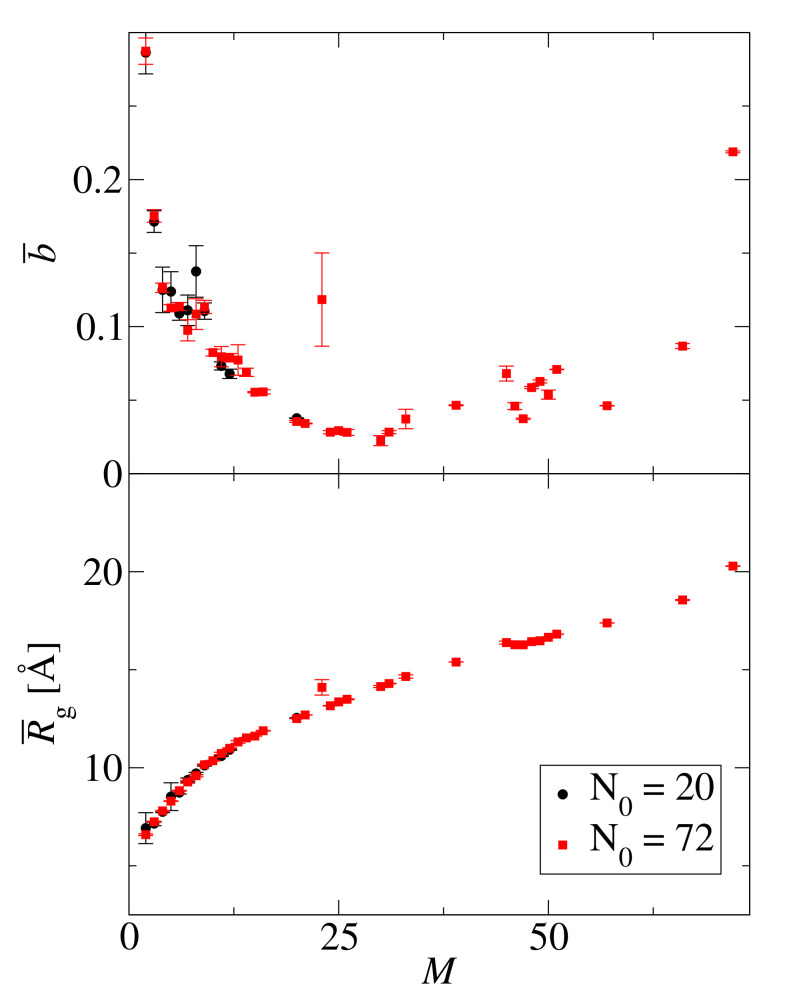
Average asphericity, $\bar{b}$ (**top panel**), and average radius of gyration, ${\bar{R}}_{g}$ (**bottom panel**), as a function of the aggregate size, *M*, for two system sizes, $N_{0}=20$ (black circles) and $N_{0}=72$ (red squares), at $c_{0}=15$ mM.

Aggregation mechanisms can be compared by plotting structural descriptors against the average cluster size *M*. Figure 3 shows the average asphericity, $\bar{b}$, and the average radius of gyration, ${\bar{R}}_{g}$, as a function of the aggregate size, *M*, for two system sizes, $N_{0}=20$ and $N_{0}=72$, at the concentration $c_{0}=15$ mM. The average radius of gyration increases with size for both system sizes. The average asphericity is also similar for both system sizes. Initially, the average asphericty drops to its smallest value $\bar{b}\approx 0.02$ at the critical aggregate size $M^{⋆}\approx 25$. After that, the asphericity grows. These results, suggest that the structural changes for GNN aggregates are independent of the size of the simulated system.

### 3.2. Fibril Formation

In our simulations, large aggregates, M> 25, show highly ordered, elongated structures. These elongated structures are helical. The GNN chains form ribbons that are twisting around the core. The fibril-like structures are formed for $N_{0}=$ 72 at all concentrations studied here, $c_{0}=$ 4–35 mM.

**Figure 4:**
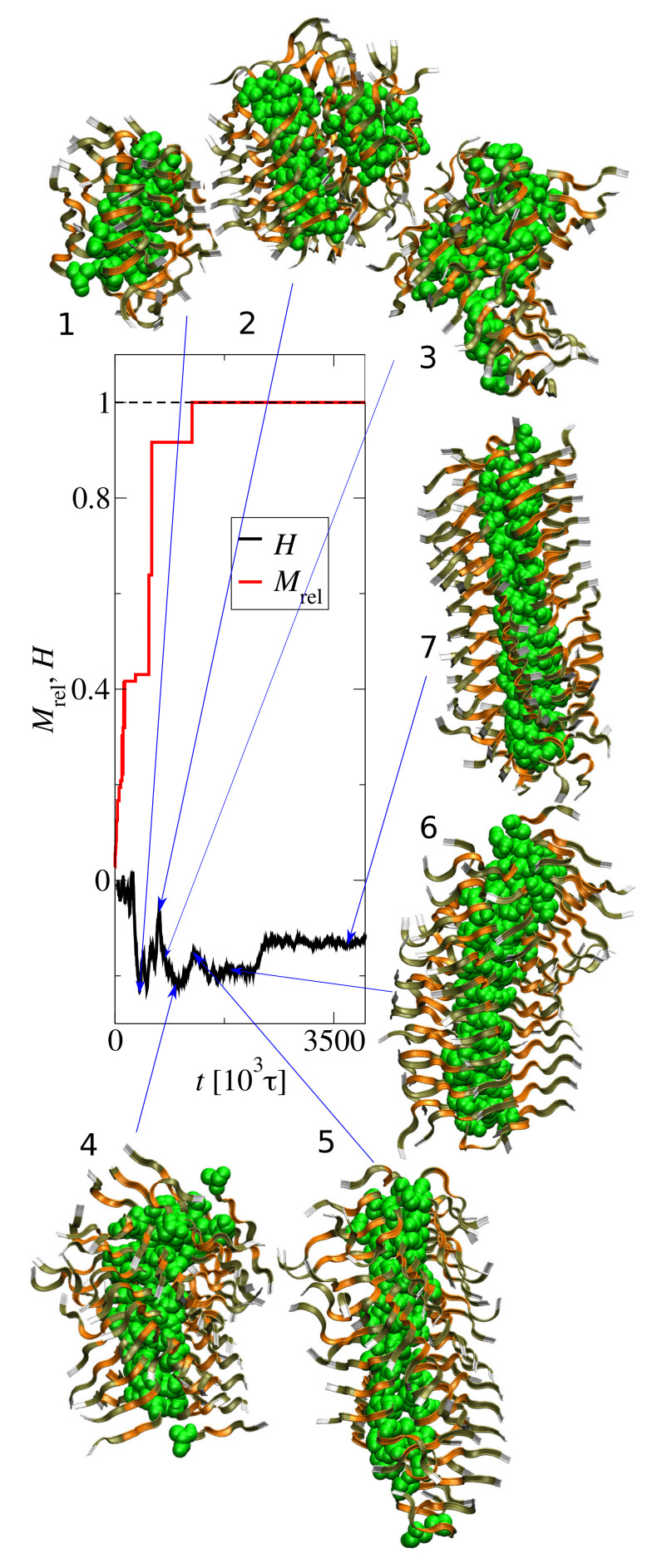
Structure evolution of the largest cluster at $N_{0}=72$ and $c_{0}=15$ mM. The helicity, *H* (black line), and the scaled size, $M_{\mathrm{rel}}=M/N_{0}$, of the largest aggregate (red line) are shown.

To describe this behavior we used the helicity descriptor *H*. Figure 4 shows the structure evolution of the largest cluster for $N_{0}=72$ and $c_{0}=15$ mM. The helicity, *H* (black line), and the scaled size of the largest aggregate, $M_{\mathrm{rel}}=M/N_{0}$ (red line), are shown. The tyrosine residues, shown as green beads, form the cluster core. The other residues are represented by ribbons. The first significant change in the helicity occurs at the aggregate size $M_{\mathrm{rel}}\approx 0.42$, which corresponds to the formation of a small fibril (snapshot 1). The coalescence of two small aggregates causes a temporary increase of the helicity up to H≈−0.07 about $t=700\times 10^{3}\tau$ (snapshot 2). This aggregate rearranges (snapshot 3), which decreases the helicity to H≈−0.22. The lowest helicity (H≈−0.22) correspond to the bent fibril presented in snapshot 4. Then the helicity increases and the fibril straightens. Around $2500\times 10^{3}\tau$, the helicity reaches a steady value of H≈−0.13 and the fibril takes an ordered, elongated structure with clearly visible ribbons twisted around the core (snapshot 7).

**Figure 5:**
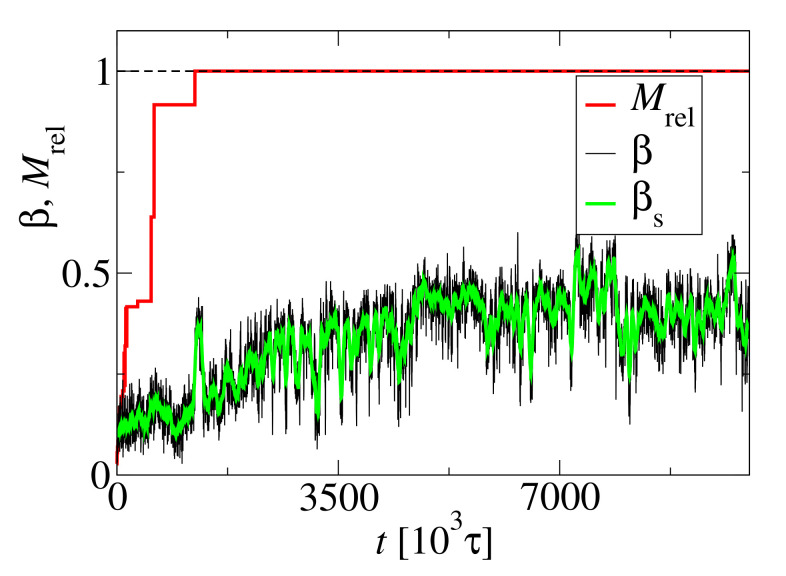
Trajectories of the beta-content, β (black line), and the scaled size of the largest cluster, $M_{\mathrm{rel}}=M/N_{0}$ (red line), for $N_{0}=72$ and $c_{0}=15$ mM. The smoothed beta-content trajectory, $\beta_{s}$, is shown as a guide to the eye.

The structural changes of clusters are connected with formation of the secondary structure. In Figure 5 an example trajectory of the beta-content, β, is shown as a black line. The size of the largest cluster *M* scaled by the number of monomers $N_{0}$, $M_{\mathrm{rel}}=M/N_{0}$, is also shown (red line) for comparison. The beta-content is close to zero for small aggregates and begins to increase when the aggregate size exceeds M=29 at about $t=1000\times 10^{3}\tau$. The high peak around $t=1200\times 10^{3}\tau$ is connected with the merging of aggregates and corresponds to the small peak on the helicity kinetic curve in Figure 4. When the size of the largest aggregate achieves its maximum value, i.e., $M=N_{0}$, the beta-content constantly increases even after the helicity achieved its steady state around $t=2500\times 10^{3}\tau$. This suggests that the peptides can further change their conformation despite that the structure of the whole fibril does not change. The beta-content begins to fluctuate around a steady value at about $t=5000\times 10^{3}\tau$.

**Figure 6:**
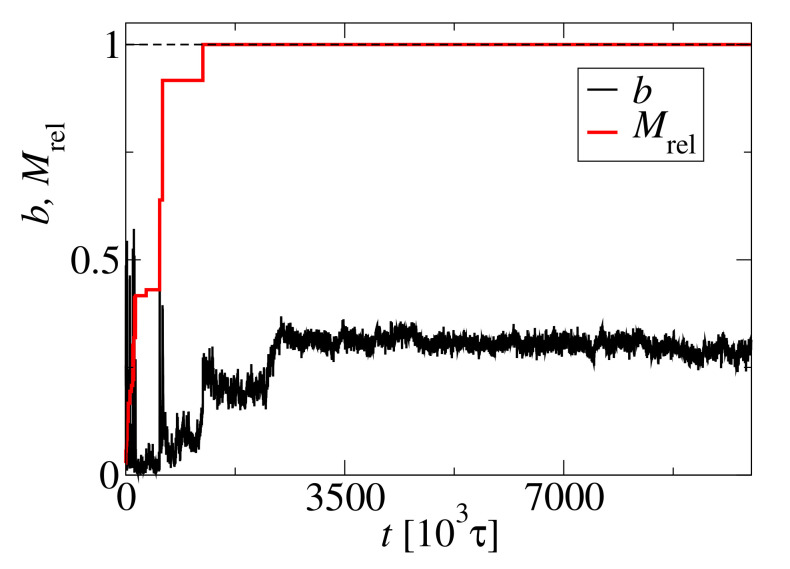
Trajectory of the asphericity, *b* (black line), for $N_{0}=72$ and $c_{0}=15$ mM. The red line showing the scaled size of the largest cluster, $M_{\mathrm{rel}}=M/N_{0}$, is the same as in Figure 5.

The fibril formation is connected with the structural transition from spherical to elongated structures. This transition can be measured by the asphericity *b*. Figure 6 shows an asphericity trajectory for the largest aggregate (black line) compared to the scaled size of the largest cluster, $M_{\mathrm{rel}}=M/N_{0}$. For small aggregates $M_{\mathrm{rel}}<0.5$, the asphericity, *b*, is close to zero, which indicates the spherical shape of small clusters. A larger aggregate formed by cluster coalescense at $t=700\times 10^{3}\tau$ transforms into a fibril-like structure. This transformation is indicated by a jump in the asphericity at $t\approx 1250\times 10^{3}\tau$. Moreover, the transformation of the final aggregate is observed as an asphericity increase at $t\approx 2250\times 10^{3}\tau$, which corresponds to an increase of the helicity *H*, see Figure 4. After this transformation, the asphericity, *b*, achives a steady value just like the helicity, *H*.

**Figure 7:**
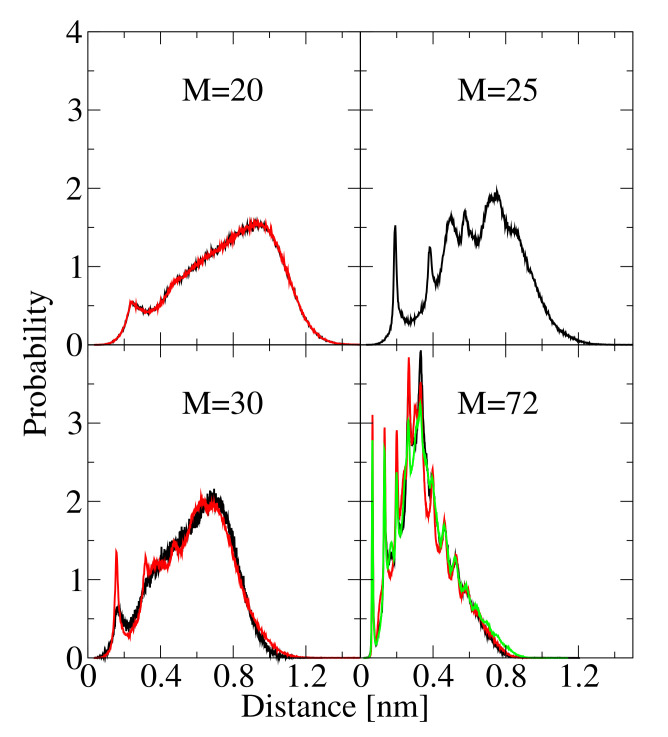
Normalized histograms of the distances between peptide mass centers in clusters for various cluster sizes: M=20 (top left panel), M=25 (top right panel), M=30 (bottom left panel) and M=72 (bottom right panel). For details see text.

The structure of the individual aggregates can be described by the mass center distance distribution. We use this descriptor to differentiate ordered from disordered clusters. Figure 7 shows the normalized histograms (probabilities) of the distances between peptide mass centers in clusters for various cluster sizes: M=20 (top left panel), M=25 (top right panel), M=30 (bottom left panel) and M=72 (bottom right panel). For M=20, the histograms from two simulations are presented: (red line) For a small system ($N_{0}=20$) at a high concentration of 35 mM, and (black line) for a larger system ($N_{0}=72$) at a low concentration of 4 mM. Despite different aggregation conditions, the aggregates are very similar. For the small system ($N_{0}=20$), an aggregate of size M=20 is the final aggregate, formed at $t\approx 100\times 10^{3}\tau$, that does not change until the end of the simulation (i.e., for about $9900\times 10^{3}\tau$). We never observed the formation of fibril-like structures for $N_{0}=$ 20. The aggregate with size M=25 is taken from a simulation for a larger system ($N_{0}=72$) at a concentration of 35 mM. Aggregates with size M=30 are presented for two simulations with $N_{0}=72$: At the concentrations 4 mM (black line) and 8 mM (red line). The largest aggregate formed for $N_{0}=72$ is presented for three simulations: Two repeats at the concentrations 35 mM (black and red lines) and 8 mM (green line).

The histograms distinguish two types of aggregates: Disordered clusters with one small peak around 0.20 nm at the first layer of neighbor peptides and a broad, fuzzy peak from other peptides. In such aggregates, there are no preferred positions of the peptide molecules in the cluster (Figure 7, top left panel). The second type of aggregates shows histograms with narrow peaks, which indicates the regular arrangement of the peptides in a cluster (Figure 7, bottom right panel). In our simulations, no aggregate with the size smaller than M=25 showed an ordered structure. The large aggregates, M>35, show the ordered structures characterized by narrow peaks on the peptide mass centers histograms. Aggregates with the size in the range 25≤M≤35 present various structures. The histogram in the top right panel of Figure 7 shows an aggregate with M=25—the smallest aggregate where we found an ordered arrangement of peptides. On the other hand, the two histograms for M=30, presented in Figure 7 right bottom panel, are different—one has the shape typical of disordered aggregates (black line) and one sports small peaks, which suggest an ordered peptide arrangement in the cluster. It is noteworthy, that the ordered histogram for M=30 (red line) has less pronounced peaks than the histogram for M=25. This difference is caused by the short lifetime for the M=30 cluster, that is about half of that for the M=25 cluster.

**Figure 8:**
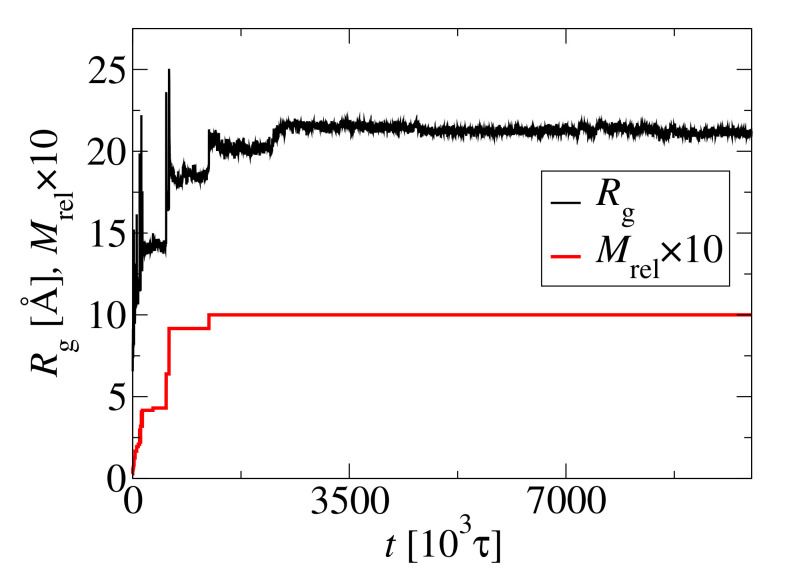
Radius of gyrations $R_{g}$ for the largest cluster (black line) and the relative size of this cluster (red line) magnified by a factor of 10, M×10, for better visualization.

The structural changes are also visible on gyration radius trajectories. Figure 8 shows the radius of gyration $R_{g}$ for the largest cluster (black line) and the relative size of this cluster (red line) multiplied by 10, 10×M, for better visualization, for $N_{0}=72$ and $c_{0}=15$ mM. The radius $R_{g}$ increases abruptly as the cluster size changes. When the final cluster is formed at $t\approx 1250\times 10^{3}\tau$, $R_{g}$ still increases to around $t=2200\times 10^{3}\tau$, which parallels the changes in the asphericity, *b* (Figure 6) and the helicity, *H* (Figure 4).

**Figure 9:**
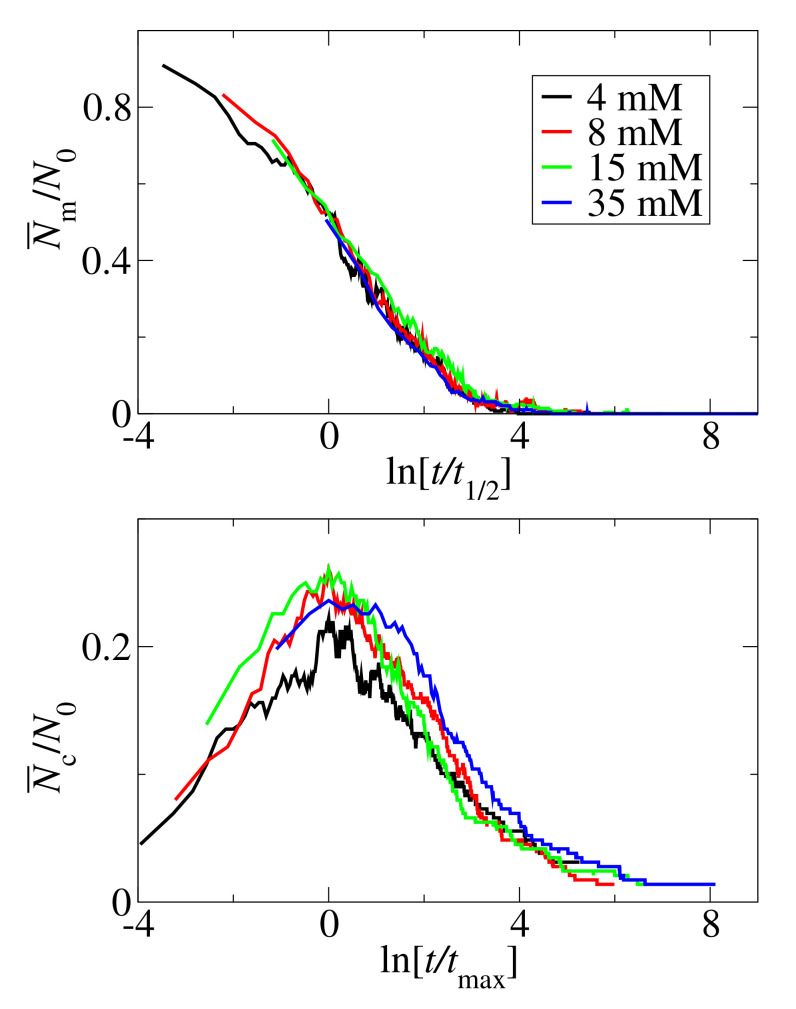
Scaled monomer (**top panel**) and clusters (**bottom panel**) kinetic curves for $N_{0}=72$ at various concentrations: $c_{0}=4$ mM (black lines), $c_{0}=8$ mM (red lines), $c_{0}=15$ mM (green lines), $c_{0}=35$ mM (blue lines).

An important question is whether the aggregation mechanism changes with concentration. Figure 9 shows the scaled monomers and clusters kinetic curves. The numbers of monomers, $N_{m}$, and clusters, $N_{c}$, are scaled by the number of peptides, $N_{0}$. For the monomer curve, time is scaled by the half-time, $t_{1/2}$. For the cluster curves, time is scaled by $t_{\max}$, defined as the time when the number of clusters reaches a maximum. The scaled kinetics curves overlap. We take this scaling property of the kinetic curves as an indication that the aggregation mechanism does not change with the concentration.

**Figure 10:**
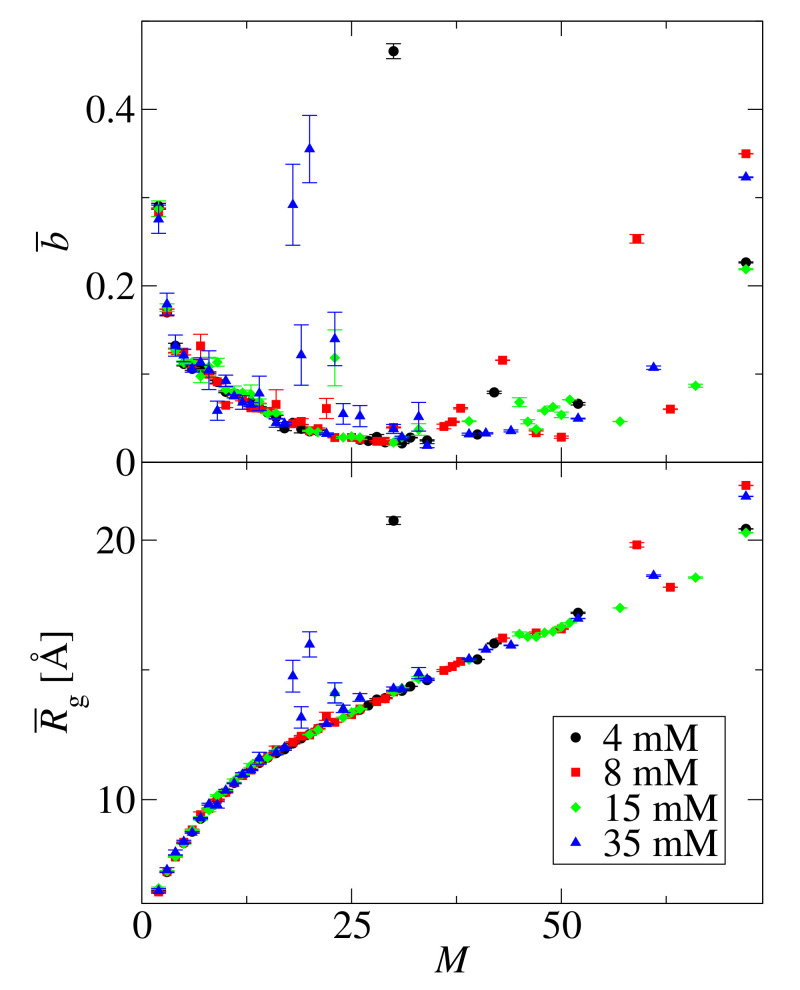
Average asphericity, $\bar{b}$ (**top panel**), and average radius of gyration, ${\bar{R}}_{g}$ (**bottom panel**), as a function of the cluster size, *M*, for concentrations $c_{0}=4$ (black circles), 8 (red squares), 15 (green diamonts), and 35 mM (blue triangles). The error bars are the standard deviations of the mean. The number of peptides $N_{0}=72$.

Figure 10 shows the average asphericity, $\bar{b}$, and the average radius of gyration, ${\bar{R}}_{g}$, as a function of the cluster size, *M*, for four simulated concentrations $c_{0}=4,8,15,35$ mM. These plots indicate that also the structural properties of clusters do not change with concentration. The average asphericity decreases initially with size and reaches its smallest value in the range 25≤M≤35. This range coincides with the structure transformation (amorphous to fibril-like) observed in the histograms of the distances between peptide mass centers, see Figure 7.

**Figure 11:**
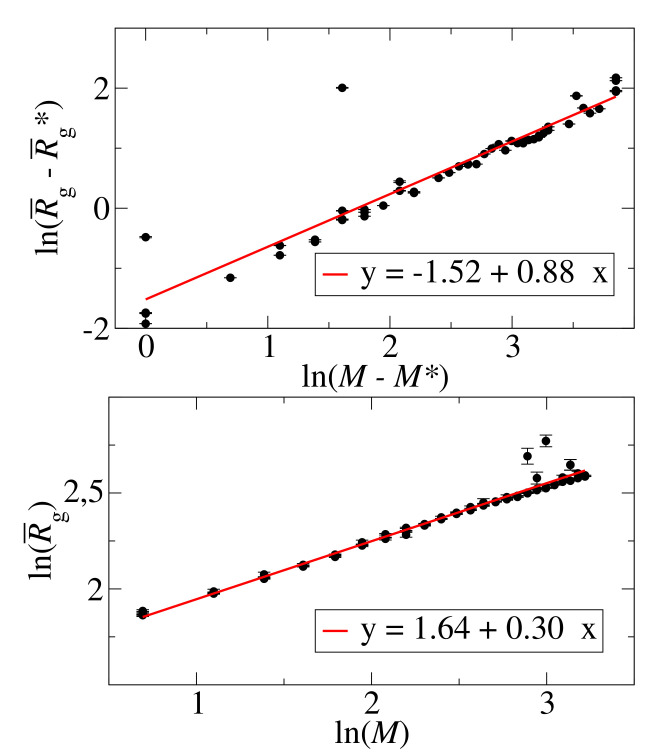
Logarithmic dependence of the average radius of gyration, ${\bar{R}}_{g}$, on the aggregate size, *M*, for small aggregates, M≤25 (**bottom panel**), and large aggregates, M≥25 (**top panel**). The radius of gyration of an aggregate with the critical size, $M^{⋆}$, is denoted as ${\bar{R}}_{g}^{⋆}$. Data for all four simulated concentrations, $c_{0}=4,8,15,35$ mM.

The average radius of gyration increases with the cluster size with a clear difference between the initial and final slopes. For small aggregates, the radius of gyration changes as

(7) $${\bar{R}}_{g}\propto M^{\alpha }$$

where α is a scaling exponent. Figure 11 shows a double logarithmic plot $\ln{\bar{R}}_{g}$ vs lnM for small aggregates, M<25. A linear fit (red line) gives the exponent α≈0.30. The radius of gyration of larger clusters changes as

(8) $${\bar{R}}_{g}-{\bar{R}}_{g}^{⋆}\propto {\left(M-M^{⋆}\right)}^{\beta }$$

where ${\bar{R}}_{g}^{⋆}=13.32$ Å is the radius of gyration of aggregates with the critical size, $M^{☆}=25$. The exponent β≈0.88 was determined by a linear fit to the double logarithmic in Figure 11. Both exponents, α and β, are lower than expected for growth in 3D and 1D, respectively. Small spherical aggregates grow in all dimensions, implying α=1/3. The lower value of α indicates that the density of cluster increases with the cluster growth. On the other hand, larger clusters, having fibril-like shape, grow by addition of monomers or small clusters to the end of a fibril. As the fibril growth is a pseudo one-dimensional process and the expected β=1. The lower value of β also suggests an increase of the aggregate density. This is consistent with the observed compaction of larger aggregates demonstrated in Figures S1 and S2 in SI.

The crossover aggregate size $M^{⋆}=25$ is the smallest aggregate size where we observe a fibril structure. We take this size as an estimate of the critical cluster size, separating small amorphous aggregates from fibril like structure. This qualitative change is supported by different scalings (7) and (8).

### 3.3. Mutation of the Tyrosine Residue

To confirm the special role of the tyrosine residue for the GNN aggregation and fibril formation we replaced the tyrosine in GNNQQNY by alanine. The mutated peptide, GNNQQNA denoted as Y7A, shows two-step aggregation. The initially formed aggregates are disordered and undergo further reorganization into a fibril-like structure. However, the Y7A fibril structure is different than that for GNN. The Y7A fibrils do not have a core and do not show helicity.

**Figure 12:**
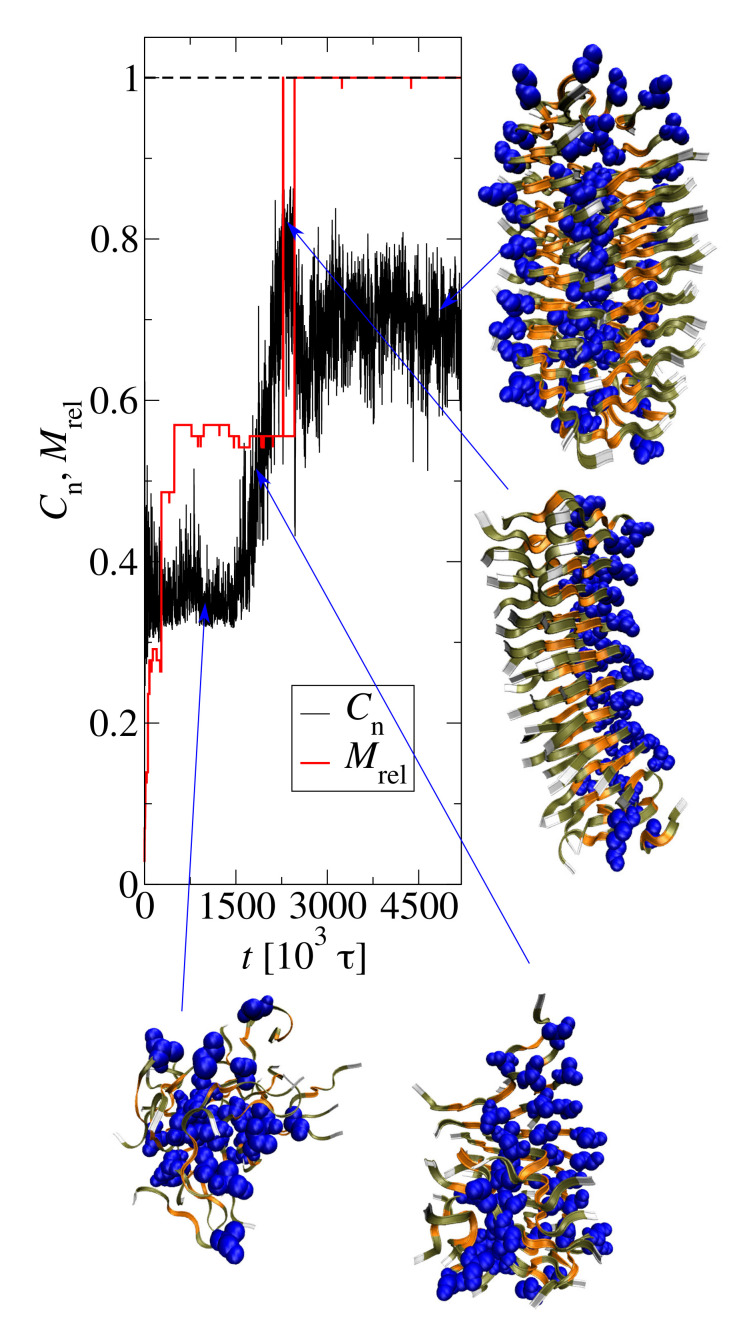
End-to-end correlation parameter, $C_{n}$, of the largest cluster as a function of time (black line) for the GNNQQNA (Y7A) aggregation. The scaled size of the largest aggregate, $M_{\mathrm{rel}}=M/N_{0}$ (red line), is shown for comparison. The evolving structures of GNNQQNA aggregates are presented as snapshots. The alanine residues are blue and highlighted using the van der Waals radius representation.

Due to the parallel peptide arrangement with no twist, the structural changes of Y7A aggregats can be captured by the end-to-end correlation parameter, $C_{n}$. Figure 12 shows an example trajectory of $C_{n}$ for the largest Y7A aggregate. The aggregate structures are also presented. The small clusters are disordered, thus $C_{n}\approx 0.33$. The aggregate with size M≈40 is initially disordered and then undergoes and internal reorganization. This reorganization is visible as an increase in $C_{n}$ at $t\approx 1600-2200\times 10^{3}\tau$. The corresponding snapshots show the formation of parallel peptide sheets. The final aggregate contains almost all peptides in peptide sheets, and the peptide sheets are parallel or antiparallel.

**Figure 13:**
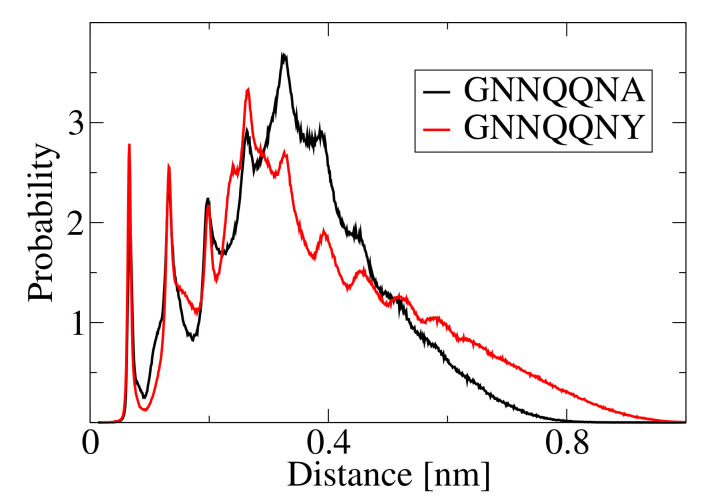
Normalized histograms of the distances between peptide mass centers in clusters: GNNQQNY (red line) and its mutation, GNNQQNA (black line).

The different peptide organization of GNN and Y7A fibrils is also visible in the histograms of the distances between peptide mass centers. Figure 13 shows the normalized histogram for GNN (red line) and Y7A (black line) final clusters. The position of the first three peaks is the same for both peptides. The shifts between the other peaks indicates the structural differences.

**Figure 14:**
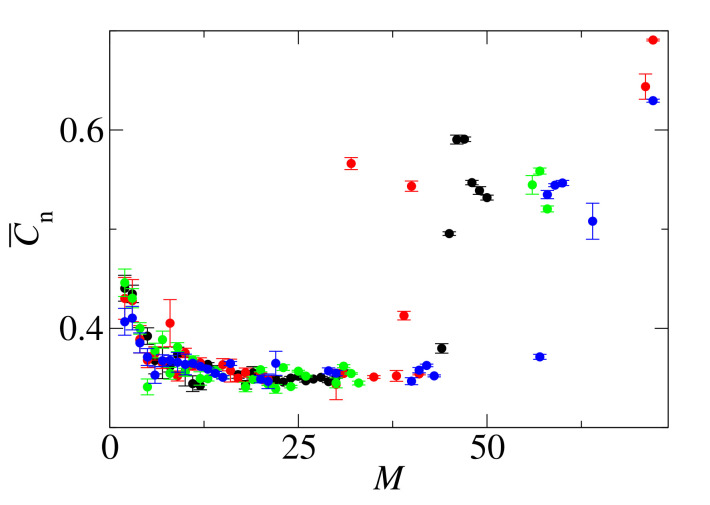
Average end-to-end correlation parameter, ${\bar{C}}_{n}$, as a function of the aggregate size, *M*, for GNNQQNA (Y7A) clusters. The four independent simulations are indicated by different colors, $N_{0}=72$ and $c_{0}=15$ mM.

Examination of the end-to-end correlation parameter, ${\bar{C}}_{n}$, as a function of aggregate size, *M*, allows for determination of the smallest aggregate with an ordered structure. Figure 14 shows $C_{n}$ as a function of *M* for four independent simulations indicated by different colors. For each repeat, the size of the smallest ordered aggregate is different: M=45 (black circles), M=32 (red circles), M=57 (green circles), M=58 (blue circles). This observation allows for estimation of the range of fibril nucleus size as *M* = 32–58. This range is higher than for GNN, *M* = 25–35.

**Figure 15:**
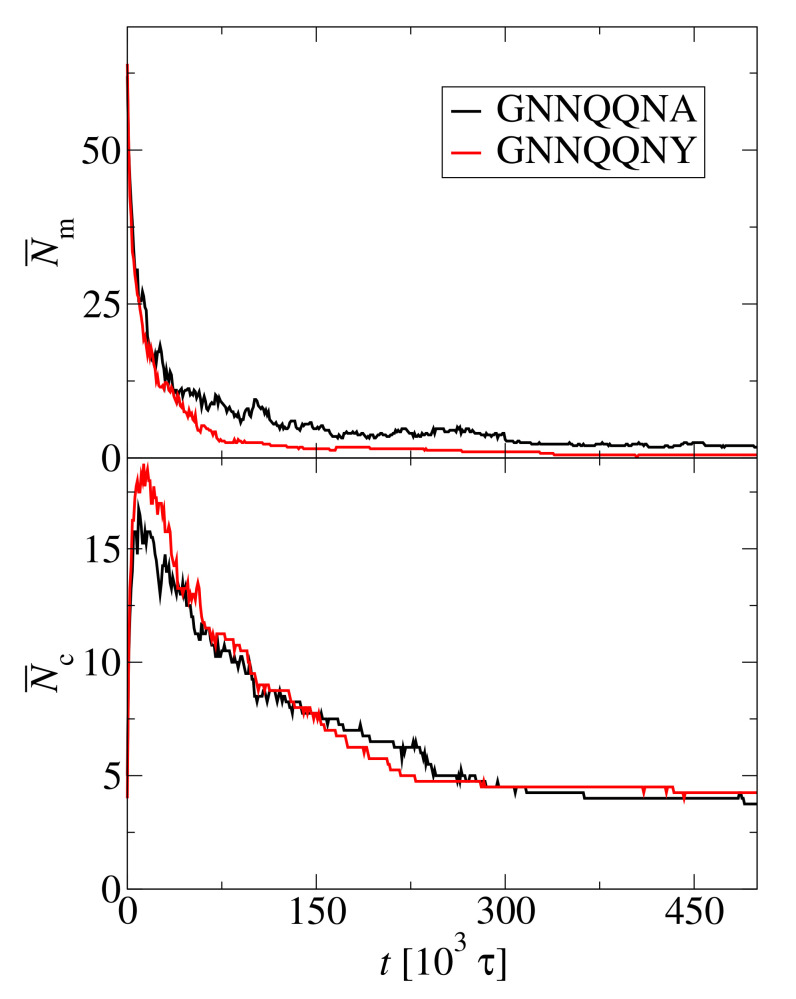
Average number of monomers, ${\bar{N}}_{m}$, (**top panel**) and average number of clusters, ${\bar{N}}_{c}$, (**bottom panel**) as a functions of time for two peptides: GNNQQNY (red line) and its mutation, GNNQQNA (black line). The averages are calculated over four simulation repeats, $N_{0}=72$ and $c_{0}=15$ mM.

The bottom panel of Figure 15 shows that the average number of clusters, ${\bar{N}}_{c}$, evolves in a similar way for GNN and Y7A. However, the decay curves for monomers are quite different as seen in the upper panel of Figure 15. Initially, the number of monomers, ${\bar{N}}_{m}$, decreases rapidly for both peptides. The difference becomes visible after the number of monomers drops below the half of initial value. The monomer decrease is slower for Y7A than for GNN. The aggregation of GNN is effectively irreversible as the number of monomers drops to zero and monomer dissociation events are not observed within the simulation time. On the other hand, the number of Y7A monomers reaches a finite steady state. After the largest aggregate is formed, the equilibrium monomer concentration, $c_{\mathrm{eq}}$ can be calculated from the remaining portion of the trajectory. For GNN $c_{\mathrm{eq}}$ is nominally 0, while for Y7A $c_{\mathrm{eq}}=0.075$ mM.

## 4. Discussion

### 4.1. Relation to Experiment

Several experimental groups studied the formation and structure of GNN aggregates and found that the aggregation propensity depends on the initial peptide concentration. Below the critical concentration (∼6 mM [29], ∼2.4 mM [10], ∼0.6 mM [11]) aggregation does not occur. Above the critical concentration aggregation is observed and various aggregate structures are formed [10,11,30]. The rate of aggregation also depends on the concentration.

For all simulated concentrations, we first observe the aggregation of monomers into small aggregates (dimers, trimers, etc.) and then the coalescence of these initial clusters into larger ones. Finally, the largest clusters assume the fibril-like structure. We found that the rate of aggregation increases with the initial concentration $c_{0}$. However, we did not find a critical concentration below which aggregation does not occur. An estimate of the critical concentration is the concentration of free monomers at equilibrium with fibrils. We found that the critical concentrations is so low that it cannot be estimated as for the concentrations $c_{0}$ = 8, 15, and 35 mM we did not see any monomers in the second halves of the trajectories.

Our simulations support the special role of tyrosine residue in the GNN aggregation [11]. The interactions between tyrosine residues are important not only for the formation of the ordered fibril-like structures, but also the formation of the initial small spherical aggregates with tyrosine cores. This is further confirmed by the behavior of the mutant Y7A.

The fibril-like clusters formed in our CG simulations are similar to the fibril model proposed by Sawaya et al. [31]. They suggested that the GNN fibrils are built from parallel β-sheets of single peptides. It is consistent with our CG simulations as the simulated fibrils contain ribbons built from parallel peptide chains. However, in the Sawaya et al. model two sheets form a pair with anti-parallel alignment of sheets and a specific steric zipper, whereas the CG fibrils have the tyrosine core and the ribbons are twisted around the core. This difference can result from the short simulated time where only the initial stage of aggregation is observed. We hypothesie that the initial fibril-like structures can further grow and transform into different inner arrangements. This is compatible with the sugestion by Marshall et al. that the observed transition from fibrils to microcrystals may envolve a rearrangement or fibril dissolution and fragmentation [11]. A second contributing factor may be the implicit solvent used in our simulations. The absence of water molecules can change the final structures of fibrils. For instance, Marshall et al. suggested that GNN fibrils, unlike crystals, include water molecules [11].

### 4.2. Comparison with Previous Simulations

All-atom GNN aggregation simulation were performed with different force fields. Srivastava and Balaji [17] used the OPLS-AA force filed to investigate the GNN aggregation in small systems ($N_{0}=$ 5, 6, 7, 8) in a 7.2 nm box. They observed the formation of the largest possible aggregate in most simulation. The large aggregates are disordered and shows loose arrangement of peptides. The β-sheet are observed for some small aggregates (dimers, trimers) and for larger ones at higher temperature. The lack of specific peptide arrangement in cluster at temperature 300 K is consistent with our simulations with the OPLS-AA model.

Nasica-Labouze and Mousseau used unbiased molecular dynamics with the OPEP coarse-grained force field to study the onset of GNN aggregation in a 20-peptide system at a concentration of 4.15 mM [22]. They suggested the critical nucleus size $M^{⋆}\approx 5$. Moreover, Nasica-Labouze and Mousseau observed a lag phase in their kinetic sigmoidal curves. In our simulations, we see downhill aggregation even at our lowest concentration of 4 mM. This difference may be attributed to the different force field and/or different cluster definition. In [22] a peptide belongs to a cluster if it is attached to another strand of that cluster by at least two hydrogen bonds.

Osborne et al. [23] studied the GNN aggregation using the BD model. They explored small systems containing 3, 6 and 12 peptides at a high concentration of 80 mM. They found the parallel peptide arrangement in clusters formed at low temperatures.

Luiken and Bolhuis [24] used the BD model to study aggregation of a 12-peptide system at a concentration of approximately 0.07 M. The order parameter for the GNN aggregation was defined as the number of in-register contacts in the largest cluster. They found that aggregation proceeds as a single step nucleation and that the size of the critical nucleus is $M^{⋆}\approx 4–6$ peptides. They also suggested the two step nucleation mechanism at higher concentrations. The present simulations confirmed this conjecture. Luiken and Bolhuis also pointed to the special role of tyrosine residues in the GNN aggregation. This is also consistent with our results.

We estimated the critical nucleus size $M^{⋆}\approx 25$ which is significantly larger than the previous estimates. Luiken and Bolhuis pointed out [24] that the estimates based on only reactive pathways do not necessarily correspond to the true critical nucleus. Here clusters are defined by a distance criterion that does not imply a reactive pathway so our estimated may be closer to the true $M^{⋆}$.

Simulation studies have also been performed for GNN mutant peptides. The substitution of tyrosine residues by alanine was investigated by all-atom simulations. Gsponer et al. found that the GNNQQNA mutant shows less kinetic stability of fibril than the wild type and that the dissaggregation events are more frequent for the mutant peptides [12]. Similarly, Zheng et al. suggest than the change of tyrosine residues decrease the fibryl stability [32]. In our simulation, the fibril stability is higher for the wild type, GNN, than for the mutant, Y7A, which is consistent with previous all-atom simulations [12,32].

### 4.3. System Size Effects

An important issue in molecular dynamics simulations is the system size effect. In our simulations, the behavior of the small $N_{0}=20$ system is qualitatively different from that for the large $N_{0}=72$ system. In the case of GNN peptides, the ordered fibril-like structures can only be observed above a critical aggregate size $M^{⋆}\approx 25$. On the other hand, the aggregation kinetics are not changed qualitatively with the system size (see the scaling plots Figure 2 and Figure 9). For all our simulations we observe the aggregation of monomers into small aggregates (dimers, trimers, etc.) and than the coalescence of these initial clusters into larger ones.

### 4.4. Concentration Effects

We performed our simulation for the concentration range $c_{0}$ = 4–35 mM. In some experimantal studies, fibrils and microcrystals were formed under such concentration [10,11]. On the other hand, other experiments report that the aggregation does not occur below ∼6 mM [29]. In our CG simulation, we observe the aggregation also at the lowest concentration 4 mM. The aggregation is faster for the higher concentrations but the mechanism and kinetics do not change. The scaled plots for large system $N_{0}=72$ show that both the monomer and cluster kinetics do not change in the studied concentration range.

## 5. Conclusions and Summary

In this work we used the BD coarse grained model to study the molecular mechanism of aggregation of the heptapeptide fragment GNNQQNY (GNN) from the yeast prion protein Sup35. We performed molecular dynamics simulations for the concentration range 4–35 mM corresponding to experimental concentrations [10,11]. By using a large system made up of $N_{0}=72$ monomers we could observe directly the formation of fibril-like structures. We introduced a new helicity descriptor *H* that quantifies the internal order of aggregates. We also found that the histogram of inter-petides distances can be useful for distinguishing between the regular and amorphous molecular arrangements.

Our simulations demnostrate that small GNN aggregates (size M<25 peptides) do not show helicity in contrast to large clusters (size M>35 peptides). This suggests that the formation of a helical structure is a cooperative process rather than an additive effect of an increasing cluster size *M*. Moreover, our simulations directly support the two-step aggregation mechanism where, in the first step, free monomers aggregate into disordered clusters and, in the second step, the large clusters reorganize into ordered structures [1]. The mechanism of fibril formation, i.e., peptide rearrangement in clusters with size M= 25–35, does not change with concentration. Thus, in the present case the two-step aggregation mechanism is not a kinetic effect of the fast initial aggregation combined with a slow internal rearrangement but rather an effect of thermodynamic/structural origin.

Our simulations support the special role of tyrosine residue in the GNN aggregation [11]. We found that the interactions between tyrosine residues are important not only for the formation of the ordered fibril-like structures but also for the formation of the initial small spherical aggregates with tyrosine cores.

In the Sawaya et al. model [31] two sheets form a pair with anti-parallel alignment and a specific steric zipper whereas the fibrils in our simulations have the tyrosine core and the ribbons are twisted around the core. This difference can result from the short simulated time where only the initial stage of aggregation is observed. We hypothesize that the initial fibril-like structures can further grow and transform into different inner arrangements.

In our simulations, the aggregation is faster at higher concentrations. The kinetic curves for monomers, dimers and clusters can be scaled to collapse on the same master curves. This suggests that the kinetics could be described by the Smoluchowski-type aggregation model which considers irreversible binary monomer and cluster aggregation. However, such a model cannot capture the difference between the small $N_{0}=20$ and large $N_{0}=72$ behavior where only clusters larger that M>25 can form fibrils. Thus, the kinetic analysis of aggregation, often used to distinguish the molecular mechanisms, may be insufficient.

## Figures and Tables

**Table 1:** GNNQQNY peptide (GNN) simulations with the Bereau and Deserno (BD) model: The initial concentration $c_{0}$, number of peptides $N_{0}$, box size *L*, and the number of simulation repeats.

$c_{0}$ [mM]	$N_{0}=20$*L* [nm]	$N_{0}=72$*L* [nm]	Repeats
4	19.57	30.00	4
8	16.31	25.00	4
15	13.05	20.00	4
35	9.79	15.00	4

## Supplementary Materials

The following are available online at https://www.mdpi.com/2218-273X/10/10/1362/s1, Figure S1: The monomer and cluster kinetic curves for the all-atom OPLS-AA force field and BD model, Figure S2: Example trajectories of the radius of gyration for the largest GNN cluster and the scaled size of the largest cluster, for the atomistic OPLS-AA force field and coarse-grained BD model, Figure S3: Average radius of gyration as a function of the aggregate size, for the OPLS-AA and BD force fields, Figure S4: Example trajectories of the asphericity, for the largest GNN cluster and the scaled size of the largest cluster for the atomistic OPLS-AA force field and the coarse-grained BD model, Figure S5: Average asphericity as a function of the aggregate size for the OPLS-AA and BD force fields, Figure S6: Example structures of the largest GNN aggregates for the atomistic OPLS-AA and coarse-grained BD force fields, Figure S7: Example histograms of the mass center distances, Figure S8: Position of the second maximum on the histogram of mass center distances as a function of the cluster size, Figures S9–11: Scaled GNN monomer and cluster kinetic curves for two system sizes at different concentrations.
