# Supplementary material for: Clustering and Fibril Formation during GNNQQNY Aggregation: A Molecular Dynamics Study
Source: Biomolecules. 2020 Sep 24;10(10):1362. doi: 10.3390/biom10101362 (PMC7598727; doi:10.3390/biom10101362)
Supplement: Supplementary file 1 [file biomolecules-10-01362-s001.pdf]

Supporting Information

for

Clustering and Fibril Formation During GNNQQNY Aggregation: a Molecular Dynamics Study

Beata Szała-Mendyk,† Andrzej Molski‡
Adam Mickiewicz University in Poznań, Faculty of Chemistry,
Umultowska 89b, 61-614 Poznań, Poland
†beata.szala@amu.edu.pl, ‡amolski@amu.edu.pl

S1. Calibration the BD time unit τ

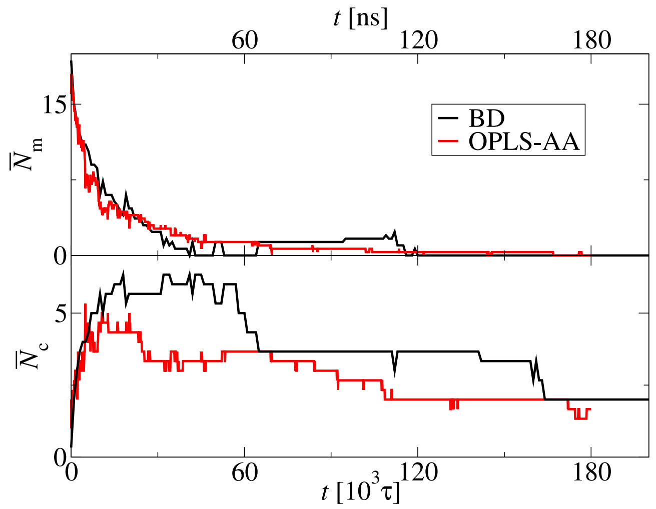

Figure S1: The monomer (top panel) and cluster (bottom panel) kinetic curves for the all-atom OPLS-AA (red lines) force field and BD model (black line) for the system size $N_0 = 20$ at the concentration c_0 =15 mM. Note the different time units: 1 ns for the OPLS-AA simulations (top axis) and $10^3\tau$ for the coarse-grained BD model (bottom axis). The kinetic curves are averages of three simulations for the OPLS-AA and four simulations for the BD model.

To calibrate the BD time unit τ we compared the all-atom OPLS-AA and coarse-grained BD kinetics for the small system, $N_0 = 20$, at $c_0 = 15$ mM. In Fig. S1 all kinetic curves are the averages of three simulations for the OPLS-AA and four simulations for the BD model. The transient OPLS-AA and BD aggregation kinetics are similar. We do not see an induction period and the monomer curves decrease right from the start of the simulations, see Fig. S1, top panel. The monomer kinetics are similar, which suggests that the coarse-grained time interval $10^3\tau$ corresponds to 1 ns for all-atom simulations. By this gauge, each BD simulation corresponds to 10 μs. The cluster kinetics curves are also similar as shown in the bottom panel of Fig. S1. The maximum number of clusters is reached at $t_{\max} = 8$ ns for OPLS-AA and $t_{\max} = 36.25 \times 10^3\tau$ for the BD model.

S2. Comparison of GNN aggregation for the OPLS-AA and BD models, $N_0 = 20$ and $c_0 = 15$ mM

The evolution of aggregate structures can be described by trajectories of the radius of gyration, R_g, and asphericity, b. Figure S2 shows example R_g trajectories (black lines) for the atomistic OPLS-AA (top panel) force field and the coarse-grained BD model (bottom panel) at $N_0 = 20$ and $c_0 = 15$ mM. For the CG trajectory only the first part of the trajectory is shown because, after the final cluster have been formed ($\approx 220 \times 10^3 \tau$), R_g only fluctuates around a steady state, $R_g = 12.5$ Å. The steady state for OPLS-AA model is higher, $R_g = 18$ Å, which corresponds to the larger size of OPLS-AA aggregate. However, the curve shapes are similar for OPLS-AA and BD models, and the numerical difference can be caused by the different sizes of individual peptides in these models. For comparison, the largest cluster size, multiplied by a factor of 10 for better visualization, is presented as a red line.

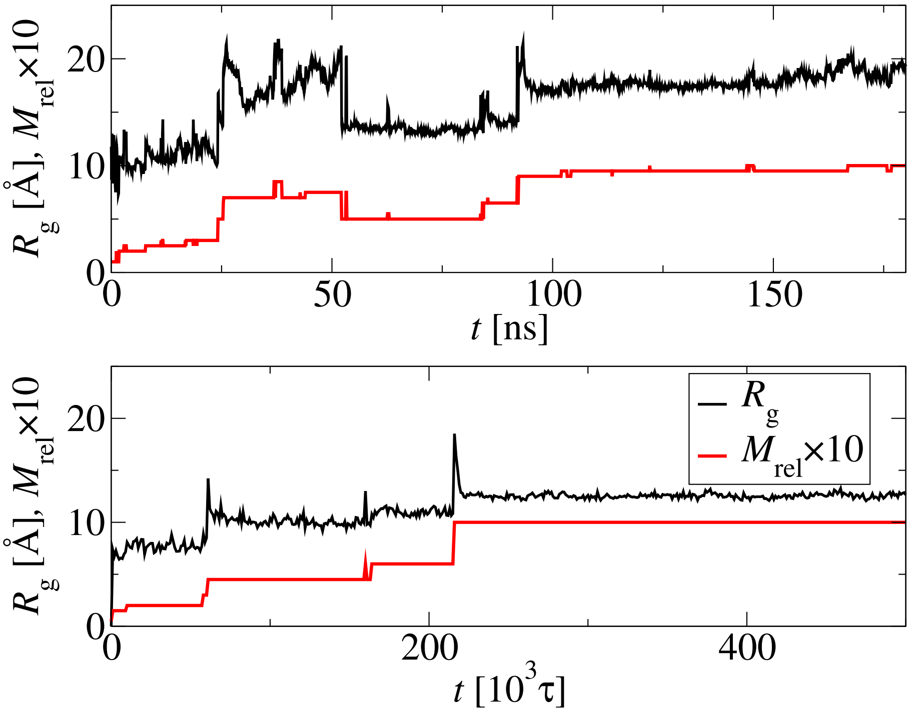

Figure S2: Example trajectories of the radius of gyration, R_g (black line), for the largest GNN cluster and the scaled size of the largest cluster, $M_{rel} = M/N_0$ (red line), for the atomistic OPLS-AA force field (top panel) and coarse-grained BD model (bottom panel).

To further compare the aggregate structures, Fig. S3 shows the average radius of gyration, $\bar{R}_g$, plotted as a function of the cluster size, M, at $N_0 = 20$ and $c_0 = 15$ mM. The structural parameters for each M were calculated as averages over all simulation repeats. The average radius of gyration scaled by the dimer radius of gyration is quite similar for both models for small aggregates up to $M \approx 10$. For larger clusters the radius of gyration is higher for the OPLS-AA clusters. This observation suggests than the OPLS-AA clusters have a lower density than the BD clusters.

Figure S4 shows example asphericity trajectories (black line) for the atomistic OPLS-AA force field (top panel) and the coarse-grained BD model (bottom panel). The scaled size of the largest GNN cluster (red line) is presented for comparison. For the CG simulation only the first part of trajectory is shown. The asphericity fluctuates around a steady state $b \approx 0.05$ since $t = 300 \times 10^3 \tau$ and does not change till the end of simulation. A small value of the asphericity corresponds to the spherical shape

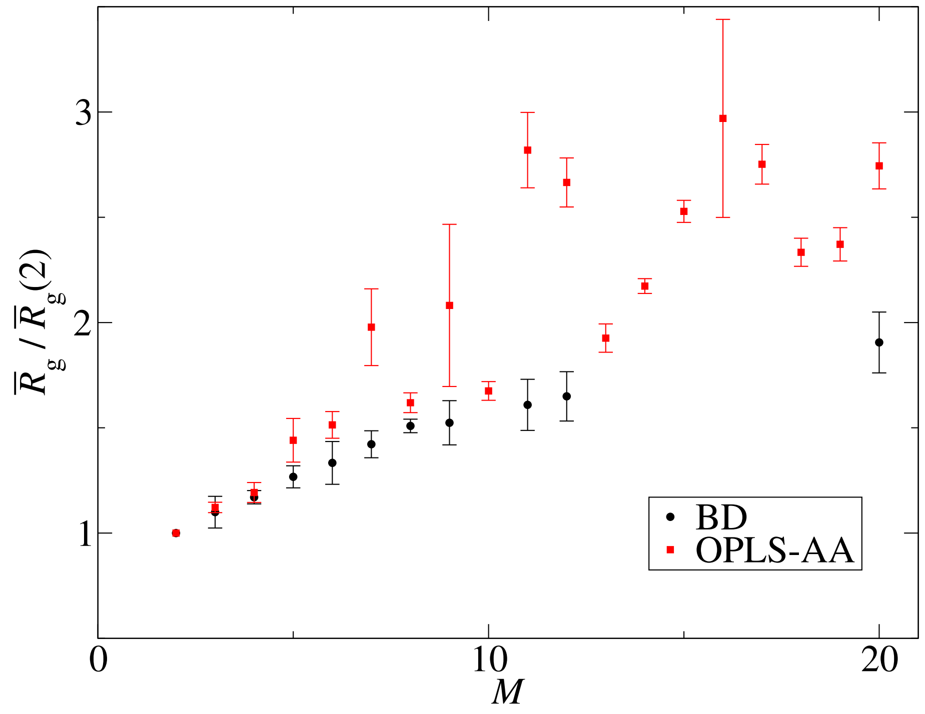

Figure S3: Average radius of gyration, $\bar{R}_{\text{g}}$, as a function of the aggregate size, M, for the OPLS-AA (red squares) and BD (black circles) force fields. For each model, the radius of gyration is scaled by its average value for dimers, $\bar{R}_{\text{g}}(2)$.

of aggregates. The asphericity trajectory is different for the OPLS-AA force field. The asphericity reaches the value $b \approx 0.45$ for the largest cluster formed at $t \approx 170$ ns.

In Fig. S5, the average asphericity, $\bar{b}$, is plotted as a function of the cluster size, M. The asphericity initially decreases for both models. However, for aggregates larger than 10, the asphericity still decreases for the BD model but increases for the OPLS-AA force field.

Fig. S6 shows example structures from the last frames of the simulations: 180 ns for OPLS-AA and $10^7\tau$ for the BD model at $c_0 = 15$ mM and $N_0 = 20$. Although both aggregates are disordered, the internal structures show differences. The OPLS-AA aggregate is loose and the peptides are randomly placed in the cluster. No preference is observed for a specific peptide arrangement. On the other hand, the CG aggregate is more dense and forms a sphere with the tyrosine residues in the center. The tyrosine interactions are observed also in experimental works and it has been suggested that the hydrophobic tyrosine interaction is one of the factors responsible for the tendency of GNN peptide to aggregate and to form fibrils [1].

The difference in the GNN aggregate structure between the atomistics OPLS-AA and CG BD force fields may be caused by the short time of all-atom simulation. In 180 ns of the OPLS-AA simulations the equilibrium state is not reached. On the other hand, the aggregation time is much longer for the BD simulations and, additionally, the aggregates undergo faster reorganization due to the structural simplification.

Different aggregate structures were observed in computational studies of GNN aggregation with various atomistics force field: OPLS-AA [2], GROMOS96 43a1 [3], PARAM22 [4], CHARMM22 [5]. Srivastava et al. [2] used the OPLS-AA force field to study GNN aggregation in small systems, $N_0 = 5, 6, 7, 8$. They observed the excess of the antiparallel peptide arrangement. Other AA and CG studies of the GNN aggregation reported various peptide arrangement in oligomers: parallel [6–9], antiparallel [10–12], and with no preference [13, 14]. This diversity of results, dependent on the AA force field used,

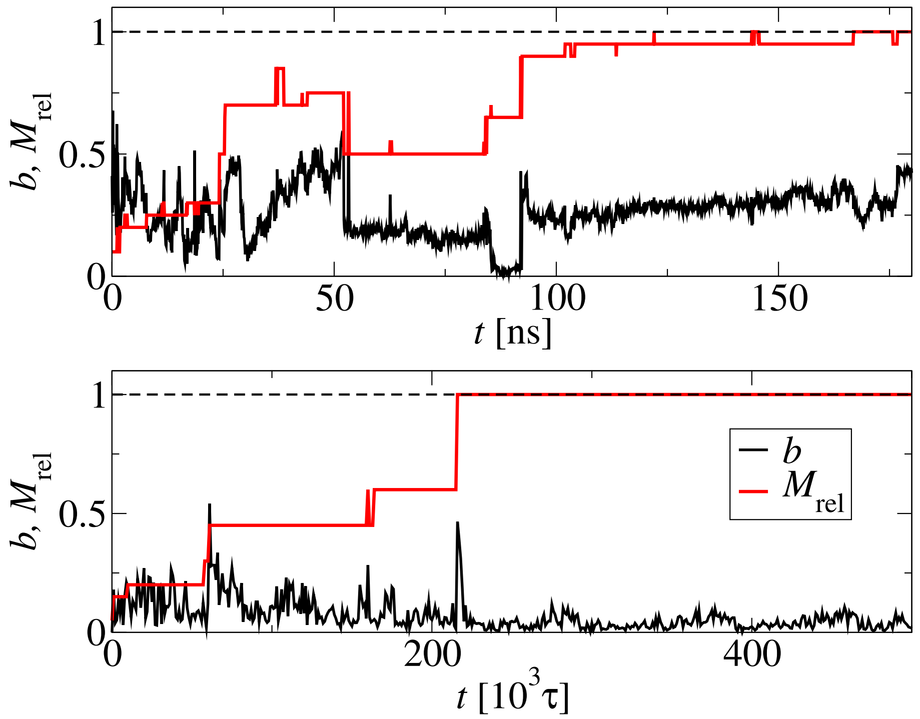

Figure S4: Example trajectories of the asphericity, b (black line), for the largest GNN cluster and the scaled size of the largest cluster,$M_{\mathrm{rel}} = M/N_0$ (red line), for the atomistic OPLS-AA force field (top panel) and the coarse-grained BD model (bottom panel).

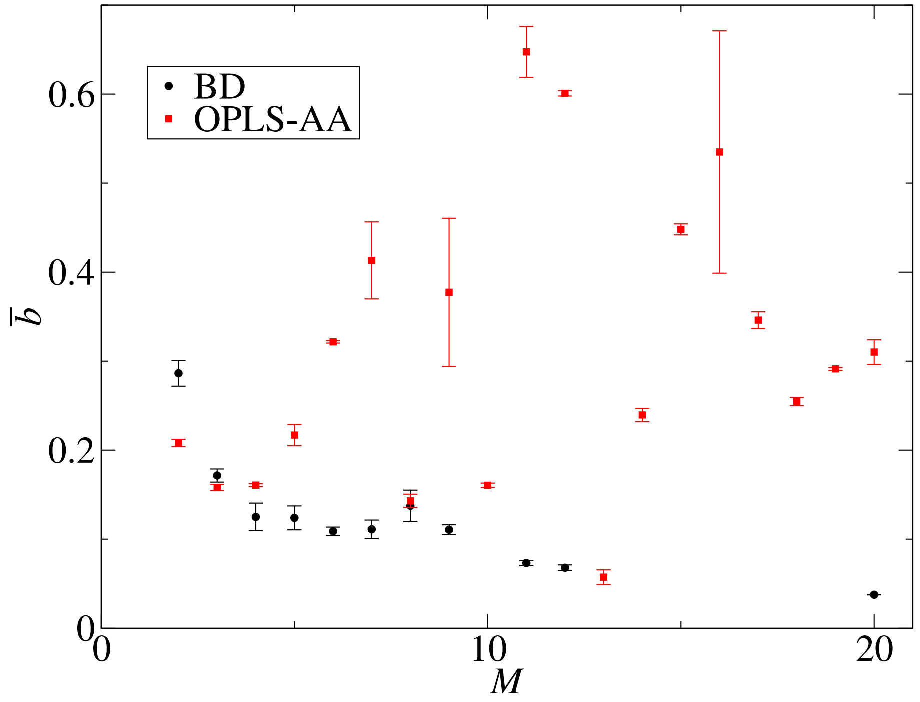

Figure S5: Average asphericity, $\bar{b}$, as a function of the aggregate size, M, for the OPLS-AA (red squares) and BD (black circles) force fields.

suggests caution when comparing transient AA and CG kinetics. For instance, it was demostrated by Strodel et al., that some AA force fields, e.g. OPLS-AA, can overestimate the protein-protein interaction leading to loss of the specificity in interactions between different amino-acids [15].

Experiments point to the important role of π-π stacking in amyloid formation [16]. Our BD simulations are consistent with a special role of tyrosine residues for the GNN fibryl formation [1].

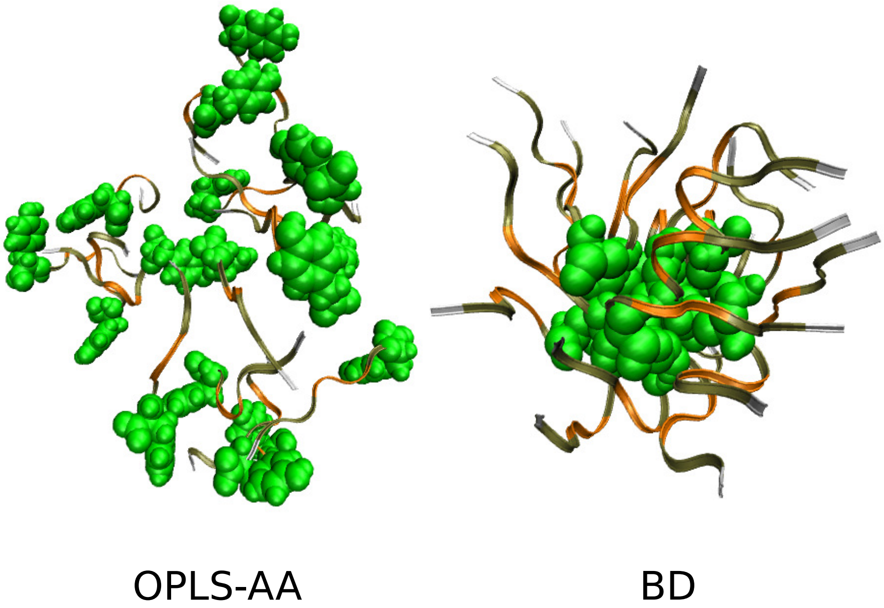

Figure S6: Example structures of the largest GNN aggregates for the atomistic OPLS-AA (left panel) and coarse-grained BD (right panel) force fields.

In this connection, we note that different AA simulations suggested different factors determining the GNN aggregation. Srivastava et al. [2] used OPLS-AA and found that the interactions between charged ends are responsible for the GNN aggregation and oligomer stability. Lei et al. suggested that the Q4 residues interaction are important as well as the side chains interactions among residues N3, Q4 and Q5 [3] for GNN aggregation in GROMOS96 43a1 force field. On the other hand, Zheng et al. used the PARAM22 force field and they found the important role of π-π interactions between tyrosine aromatic rings [4]. Gsponer et al. used the PARAM19 force field and observed that the in-register parallel aggregates are stabized by the side-chain hydrogen bonds and the aromatic interactions between tyrosine resudues [6].

S3. Compaction of BD aggregates as their size increases

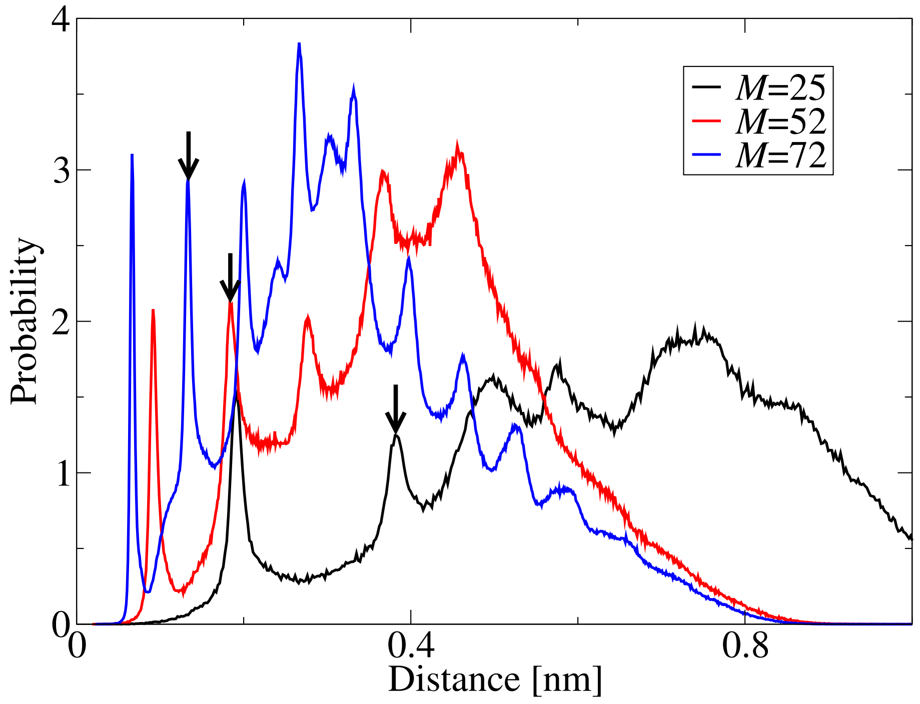

Figure S7: Example histograms of the mass center distances for clusters of size $M = 25$ (black line), $M = 52$ (red line), and $M = 72$ (blue line). The position of the second maximum changes with the cluster size. The black arrows mark the second maximum at ≈ 0.38 nm for $M = 25$, at ≈ 0.19 nm for $M = 52$, and at ≈ 0.13 nm for $M = 72$.

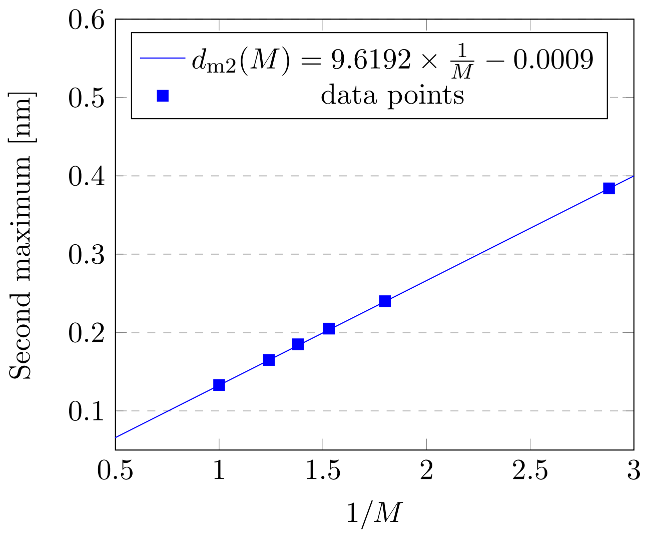

Figure S8: Position of the second maximum on the histogram of mass center distances as a function of the cluster size M. The position of the second maximum for a cluster size M is the average over all aggregates of size M from all simulations. The squares indicate the simulation data and the line is the linear regression fit.

S4. Scaled GNN aggregation kinetics for $N_0 = 20$ and $N_0 = 72$ are similar

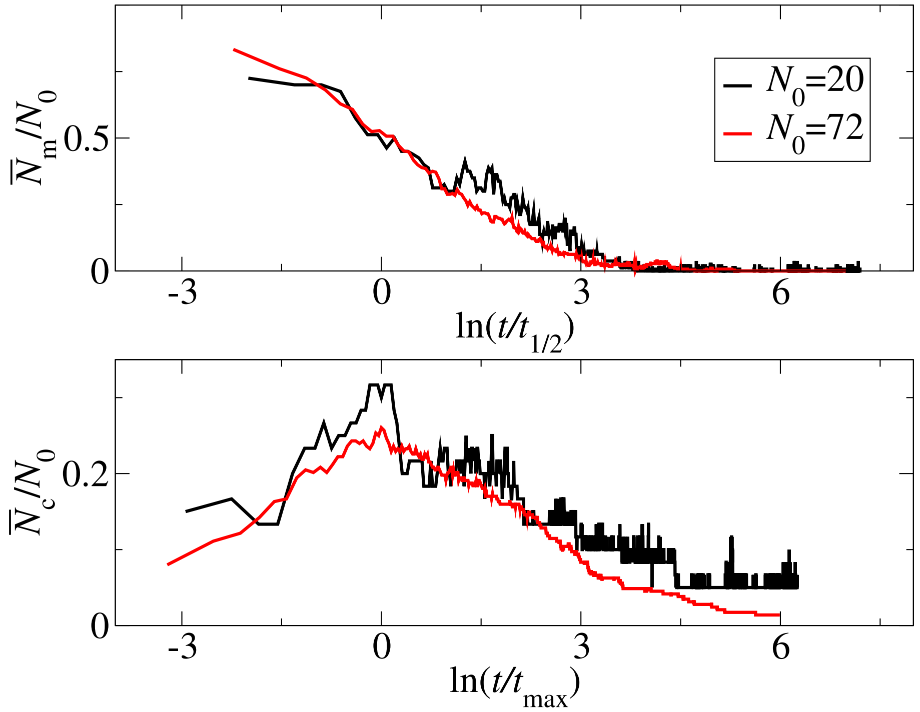

Figure S9: Scaled GNN monomer (top panel) and cluster (bottom panel) kinetic curves for two system sizes, $N_0 = 20$ (black lines) and $N_0 = 72$ (red lines), at the concentration $c_0 = 8$ mM. The number of monomers, N_m, and the number of clusters, N_c, are scaled by the initial number of peptides, N_0. For the monomer curves, time is scaled by the monomer decay half-time, $t_{1/2}$. For the cluster curves, time is scaled by t_{max}, defined as the time when the number of clusters reaches its maximum value.

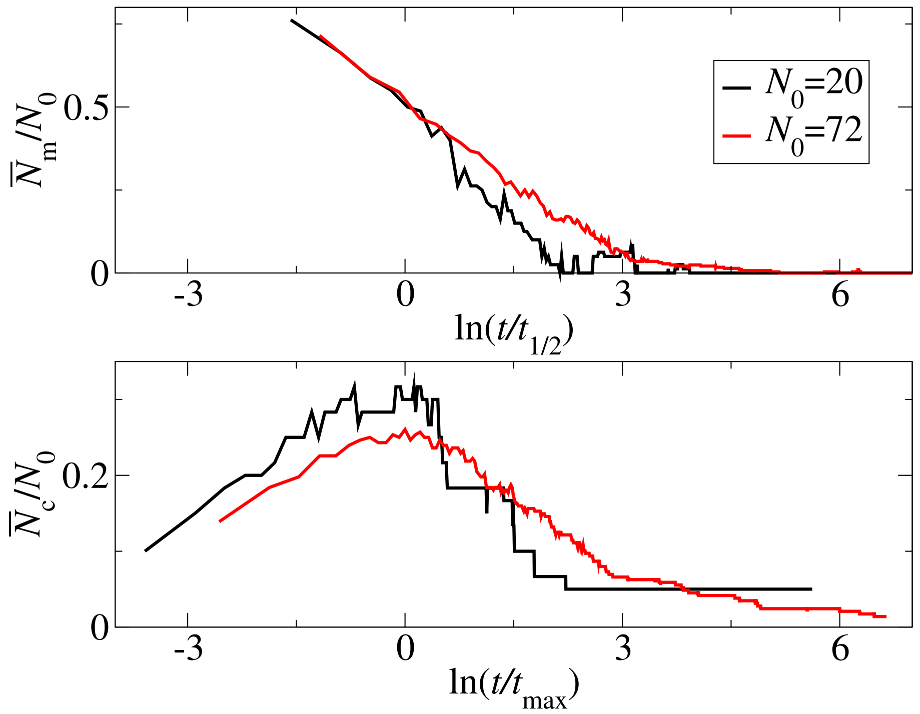

Figure S10: Same as in Fig. S9 except that the concentration $c_0 = 15$ mM.

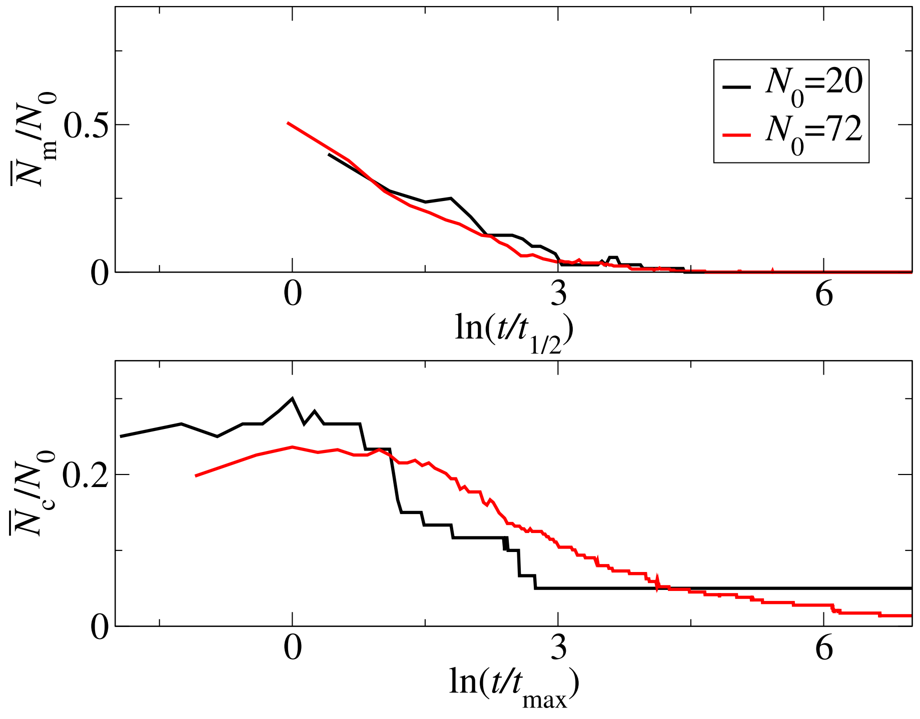

Figure S11: Same as in Fig. S9 except that the concentration $c_0 = 35$ mM.

References

[1] K. E. Marshall, M. R. Hicks, T. L. Williams, S. V. Hoffmann, A. Rodger, T. R. Dafforn, and L. C. Serpell. Characterizing the assembly of the Sup35 yeast prion fragment, GNNQQNY: Structural changes accompany a fiber-to-crystal switch. *Biophys. J.*, 98:330–338, 2010.

[2] A. Srivastava and P. V. Balaji. Molecular events during the early stages of aggregation of GNNQQNY: An all atom md simulation study of randomly dispersed peptides. *J. Struct. Biol.*, 192:376–391, 2015.

[3] J. Lei, R. Qi, L. Xie, W Xi, and G. Wei. Inhibitory effect of hydrophobic fullerenes on the beta-sheet-rich oligomers of a hydrophilic GNNQQNY peptide revealed by atomistic simulations. *RSC Adv.*, 7:13947–13956, 2017.

[4] J. Zheng, B. Ma, C.-J Sai, and R. Nussinov. Structural stability and dynamics of an amyloid-forming peptide GNNQQNY from the yeast prion Sup-35. *Biophys. J.*, 91:824–833, 2006.

[5] J. Lipfert, J. Franklin, F. Wu, and Doniach S. Protein misfolding and amyloid formation for the peptide GNNQQNY from yeast prion protein sup35: Simulation by reaction path annealing. *J. Mol. Biol.*, 349:648–658, 2005.

[6] J. Gsponer, U. Habertuer, and A. Caflish. The role of side-chain interactions in the early steps of aggregation: Molecular dynamics simulations of an amyloid-forming peptide from the yeast prion Sup35. *Proc. Natl. Acad. Sci. U.S.A.*, 100:5154–5159, 2003.

[7] M. Meli, G. Morra, and G Colombo. Investigating the mechanism of peptide aggregation: insights from mixed monte carlo-molecular dynamics simulations. *Biophys. J.*, 94:4414–4426, 2008.

[8] K. L. Osborne, M. Bachmann, and Strodel B. Thermodynamic analysis of structuredal transitions during GNNQQNY aggregation. *Proteins*, 81:1141–1155, 2013.

[9] B. Barz, D. J. Wales, and B. Strodel. A kinetic approach to the sequence-aggregation relationship in disease-related protein assembly. *J. Phys. Chem. B*, 118:1003–1011, 2014.

[10] Z. Zhang, H. Chen, H. Bai, and L. Lai. Molecular dynamics simulations on the oligomer-formation process of the gnnqqny peptide from yeast prion protein sup35. *Biophys. J.*, 93:1484–1492, 2007.

[11] J. Nasica-Labouze, M. Meli, and P. Derreumaux. Approach to characterize the early aggregation steps of the amyloid-forming peptide GNNQQNY from the yeast prion Sup-35. *PLoS Comput. Biol.*, 7:e1002782, 2011.

[12] J. Nasica-Labouze and N. Mousseau. Kinetics of amyloid aggregation: A study of the GNNQQNY prion sequence. *PLoS Comput. Biol.*, 8:e1002782, 2012.

[13] M. Cecchini, F. Rao, M. Seeber, and A. Caflisch. Replica exchange molecular dynamics simulations of amyloid peptide aggregation. *J. Chem. Phys.*, 21:10748–10756, 2004.

[14] B. Strodel, C. S. Whittleston, and Wales D. J. Thermodynamics and kinetics of aggregation for the GNNQQNY peptide. *J. Am. Chem. Soc.*, 129:16005–16014, 2007.

[15] M. Carballo-Pacheco, A. E. Ismail, and B. Strodel. On the applicability of force fields to study the aggregation of amyloidogenic peptides using molecular dynamics simulations. *J. Chem. Theory Comput.*, 14:6063–6075, 2018.

[16] O. S. Makin, E. Atkins, P. Sikorski, J. Johansson, and L. C Serpell. Molecular basis for amyloid fibril formation and stability. *Proc. Natl. Acad. Sci. U.S.A.*, 102:315–320, 2005.
